# Protein Composition and Postprandial Aminoacidemia of Biscuits Fortified with Brewer’s Spent Grain and Fermented Brewer’s Spent Grain in Individuals with Metabolic Syndrome

**DOI:** 10.3390/foods15193558

**Published:** 2026-10-07

**Authors:** Zi Ning Leong, Yujing Xu, Jialin Chen, Yidan Chang, Gregory Chung Tsing Chan, Jung Eun Kim

**Affiliations:** 1 Department of Food Science and Technology, Faculty of Science, National University of Singapore, Singapore 117543, Singapore; leong.zining@u.nus.edu (Z.N.L.); yujingxu@u.nus.edu (Y.X.); e1124656@u.nus.edu (J.C.); chang_yidan_@u.nus.edu (Y.C.); 2 Saw Swee Hock School of Public Health, National University of Singapore, Singapore 117549, Singapore; ephgchan@nus.edu.sg; 3 Bezos Center for Sustainable Protein, National University of Singapore, Singapore 117542, Singapore

**Keywords:** Brewer’s spent grain, *Rhizopus oligosporus*, amino acid score, postprandial amino acid response, metabolic syndrome

## Abstract

Brewer’s spent grain (BSG), the main side-stream of beer brewing, is an abundant and low-cost source of plant protein with limited digestibility. Fermentation pre-treatment may improve protein quality by altering amino acid composition and increasing protein accessibility. This study investigated whether the solid-state fermentation of BSG with *Rhizopus oligosporus* could improve amino acid composition and further elicit a greater postprandial amino acid response after the consumption of BSG-incorporated biscuits in adults with metabolic syndrome (MetS). Control biscuits (100% wheat flour) and biscuits with a 30% substitution of autoclaved BSG and fermented BSG were prepared. Compositional improvements were evaluated via an analysis of total and free amino acids and the amino acid score calculated against adult indispensable amino acid requirements. Fifteen adults with MetS then consumed 90 g of each biscuit in a randomised, double-blind, crossover trial, with postprandial plasma amino acids assessed over 120 min. Incorporating BSG increased the total amino acid content in biscuits, while fermentation further increased free amino acid content. The lysine amino acid score also increased from 0.57 (Control) to 0.81 (fermented BSG). Despite these compositional improvements, postprandial total and essential amino acid responses did not differ between biscuits, indicating a disconnect between improved protein composition and postprandial amino acid responses in individuals with MetS.

## 1. Introduction

Brewer’s spent grain (BSG) is the main side-stream generated after wort production during beer brewing, with about 20 kg of BSG produced for every 100 L of beer [1,2]. Consisting mainly of barley grain shells and smaller fractions of the pericarp and endosperm layers of barley [3], BSG contains 70% lignocellulose matter [4] and around 20% crude protein content, as well as other biologically active compounds including phenolic compounds [5], making it a low-cost, abundant food-grade byproduct that is full of untapped nutritional potential [6]. Consequently, the incorporation of BSG into food products has attracted increasing interest as a potential way to reduce food waste while promoting a sustainable, alternative plant-based protein source.

From a nutritional perspective, BSG is a promising complementary plant protein source. Although its protein content is lower than that of soy (33–50%), it presents a comparable amount of protein to other alternative plant protein sources such as pea and faba beans (16–29%) [7]. Besides total protein content, the amino acid composition, digestibility and post-digestion bioavailability of essential amino acids also affect protein quality [8,9]. In this regard, compared to other cereal-derived proteins, BSG contains relatively higher levels of lysine [6], which is commonly noted as one of the limiting amino acids present in most plant-based diets [10,11]. Pérez-Alva et al. [12] note that BSG may contain up to 3–4 times more lysine than wheat flour. However, the digestibility of BSG proteins is still limited due to the abundance of hordeins [13], anti-nutrients [14] and the recalcitrant cell structure [15]. Most proteins that remain in BSG after the mashing process of beer-making are difficult to break down as they have formed enhanced disulfide crosslinks and aggregation. As a result, although BSG contains nutritionally valuable amino acids, the digestibility and effective utilisation of these proteins may be limited if used without pre-treatment [16].

Among other pre-treatment methods, solid-state fermentation using filamentous fungi, such as *Rhizopus oligosporus* (RO), has gained attention for its ability to improve the nutritional properties of BSG. Previous studies have reported increases in soluble fibre, nitrogen and free amino acid contents, alongside reductions in anti-nutritional factors, suggesting that fermentation may improve protein quality and nutrient availability [17,18,19]. However, most studies evaluating fermented BSG have focused primarily on compositional changes or in vitro protein digestibility [4], and whether these improvements translate into further physiological benefits in humans have yet to be established.

In humans, the nutritional value of dietary protein ultimately depends on the extent to which amino acids are released during digestion, absorbed across the intestinal epithelium, and made available in the systemic circulation for metabolic utilisation. Consequently, while postprandial plasma amino acid responses (PPARs) cannot directly measure absolute exogenous availability in the absence of tracers, the magnitude of the response can characterise how amino acids from different food sources appear in the circulation over time following ingestion, confirming that dietary protein has been digested and absorbed [20,21]. Therefore, the present study aimed to firstly evaluate changes in the amino acid composition of wheat biscuits partially substituted with autoclaved or RO-fermented BSG, followed by assessing PPAR after biscuit consumption in individuals with metabolic syndrome (MetS). As the dietary management of MetS often encourages the greater consumption of plant-derived proteins [22,23], evaluating the metabolic availability of amino acids from emerging plant protein sources such as BSG is particularly relevant for this population. It is hypothesised that the fermentation of BSG would increase protein accessibility and free amino acid content, resulting in a greater postprandial plasma amino acid response compared with biscuits containing unfermented BSG in individuals with MetS.

## 2. Materials and Methods

### 2.1. Biscuit Preparation

The fermentation of BSG, the preparation of the biscuits and their nutritional information were previously published in detail in Wang et al. [24]. Briefly, BSG from Brewerkz Brewing Co. (Singapore) was sterilised at 121 °C for 15 min to produce autoclaved BSG (HICLAVE HG-80, Hirayama, Saitama, Japan). Fermented BSG was obtained by the fungal fermentation of autoclaved BSG with 4% of ragi tempe starter (PT. Aneka Fermentasi Industri, Bandung, Indonesia) at 37 °C for three days. Both autoclaved and fermented BSG were freeze-dried (Lyovapor L-300, BÜCHI, Flawil, Switzerland) to a moisture content of less than 5% and stored in sealed Ziploc bags at −20 °C until use. They were ground (EGK 200, Rommelsbacher ElektroHausgeräte GmbH, Dinkelsbühl, Germany) to autoclaved BSG powder (AP-BSG) and fermented BSG powder (FP-BSG) to be used as flour. 

Three types of biscuits, plain biscuits without BSG (Control biscuits), biscuits with 30% AP-BSG (ABSG biscuits), and biscuits with 30% FP-BSG (FBSG biscuits), were prepared. In the ABSG and FBSG formulations, BSG flour replaced 30% of 180 g wheat flour used in the Control biscuit formulation. The remaining ingredients were identical across all three formulations, comprising 50 g icing sugar, 70 g whole egg, 55 g sunflower oil, 1 g baking soda, 1 g salt and 10 g corn flour. All biscuits were baked at 160 °C for 20 min (Anna, Unox, Cadoneghe, Italy). For ease of reference, detailed nutritional information about the biscuits and flour as described in previous works was retabulated for 90 g (biscuits) and 100 g servings (flours) and are presented in Table 1.

### 2.2. Amino Acid Analysis of Biscuits and Powder

The amino acid profiles of the test biscuits (ABSG, FBSG, and Control) and flours (AP-BSG and FP-BSG) were determined using an amino acid analyser (LA8080, Hitachi, Tokyo, Japan). Total amino acid (TAA) content was determined through acid hydrolysis (6N hydrochloric acid, 110 °C, 24 h under nitrogen). Tryptophan was recovered by alkaline hydrolysis (5N sodium hydroxide, 110 °C, 24 h under nitrogen), and cysteine and methionine were recovered following a 20 h pre-oxidation step with performic acid prior to acid hydrolysis. This yielded a total quantification of 18 standard amino acids, namely alanine, arginine, aspartic acid, cysteine, glutamic acid, glycine, histidine, isoleucine, leucine, lysine, methionine, phenylalanine, proline, serine, threonine, tryptophan, tyrosine and valine. Asparagine and glutamine, the remaining two amino acids of the twenty proteinogenic amino acids, were not quantified as they were converted to aspartic acid and glutamic acid during acid hydrolysis. Analysis was conducted in triplicates, with the highest concentration of amino acids from each of the three hydrolysis methods being used to calculate the TAA content in the biscuits. All chemicals and reagents were purchased from Sigma-Aldrich (St. Louis, MO, USA) unless otherwise stated.

The free amino acids (FAAs) of the biscuits and flours were determined after defatting. A total of 0.5 g of samples was vortex-mixed (Vortex-Genie 2, Scientific Industries, Inc., Bohemia, NY, USA) with 5 mL of hexane for 10 min, with the upper hexane layer discarded and residue dried at 55 °C to produce defatted samples. FAA was then extracted by vortex-mixing 0.2 g of the defatted samples with 0.8 mL of 0.02 N HCl for 5 min. Following centrifugal separation (15,000× *g*, 5 min), the supernatant was mixed with 0.15 mL of 10% trichloroacetic acid (TCA) and left to stand at 4 °C for an hour. The resulting supernatant was centrifuged (11,000× *g*, 15 min) and filtered through a 0.22 µm PTFE filter before analysis.

### 2.3. Amino Acid Score

The amino acid scores (AASs) of the biscuits were calculated by comparing indispensable amino acid contents expressed as mg/g protein with the Food and Agriculture Organization of the United Nations (FAO) adult indispensable amino acid reference pattern [25]. The AAS was calculated as the ratio between the amino acid content of the test sample and the corresponding amino acid requirement in the reference pattern to identify the limiting amino acid.

$$AAS=\frac{\mathrm{mg}\;\mathrm{indispensable}\;\mathrm{amino}\;\mathrm{acid}\;\mathrm{per}\;g\;\mathrm{protein}\;\mathrm{in}\;\mathrm{sample}}{\mathrm{mg}\;\mathrm{indispensable}\;\mathrm{amino}\;\mathrm{acid}\;\mathrm{per}\;g\;\mathrm{protein}\;\mathrm{in}\;\mathrm{FAO}\;\mathrm{reference}}$$

### 2.4. Clinical Study Procedures

The study protocol was approved by the National University of Singapore Institutional Review Board (NUS-IRB-2022-089) and registered at clinicaltrials.gov (NCT05421780). The detailed recruitment criteria, CONSORT flow diagram and study procedures can be found in Xu et al. [26]. In short, participants aged 35–85, who met at least three out of five of the following MetS criteria in accordance with National Cholesterol Education Program Adult Treatment Panel III (NCEP-ATP III), were recruited for this study. In total, sixteen participants were recruited, with one dropping out due to personal reasons. A total of fifteen participants completed this study.

**Figure 1:**
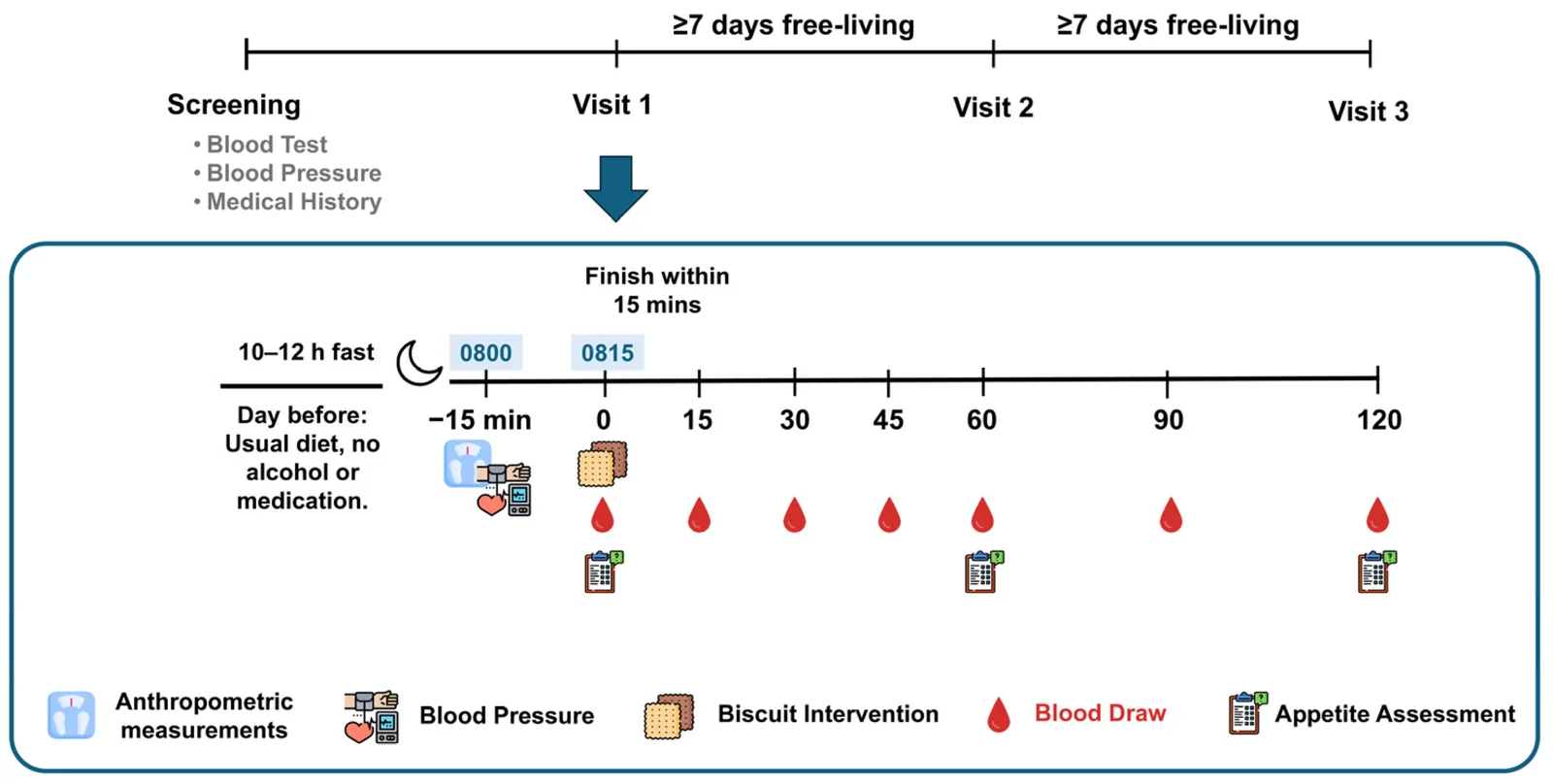
Schematic diagram of study visits, where procedures common to all visits are detailed by the arrow, with Visit 1 as an example. Icons in legend indicate the procedures conducted at each timepoint.

This study was a double-blind, crossover study with a one-week washout period in between visits. A study visit schematic is shown in Figure 1. Briefly, participants arrived after an overnight fast, and anthropometric measurements and blood pressure were first taken prior to them lying down in a phlebotomy bed for the duration of the visit. Blood samples were collected via an indwelling venous cannula inserted into the forearm. After fasting blood was taken (time = 0 min, T0), participants consumed one randomly assigned pack of biscuits (90 g) with plain water. The timer was started on their first bite, and subsequent blood samples were collected at the timepoints shown in Figure 1.

### 2.5. Blood Sample Analysis Preparation

Plasma FAA concentrations were determined similarly to those in biscuits and powder, with 10% TCA being added in a ratio of 1:4 to defrosted blood plasma samples and samples being left for one hour before centrifuging. After this, the supernatant was filtered with a 0.22 µm PTFE filter before analysis. All blood sample analyses were conducted in duplicates with samples prepared no earlier than three days before analysis.

### 2.6. Postprandial Plasma Amino Acid Response (PPAR) Analysis

To examine changes in PPAR, both time course and kinetic parameters after the intervention were determined. The plasma TAA (P-T21AA) panel comprise the 20 standard amino acids, as well as taurine, a specific non-proteinogenic amino acid that is classified as non-essential. Plasma essential amino acid (P-EAA) and plasma non-essential amino acid (P-NEAA) groupings are established from Hou et al. [27].

#### 2.6.1. Postprandial Time Course Analysis

The time course changes in individual amino acid concentrations in the plasma over the 120 min postprandial period were analysed using an analysis of covariance (ANCOVA)-style linear mixed model (LMM). Non-baseline-corrected concentration data was used for the analysis, with the fasting concentration (T0) used as a fixed covariate within the model. The model was structured as follows:

$$Y_{ijkt}=\beta_{0}+\beta_{1}{\mathrm{Intervention}}_{j}+\beta_{2}{\mathrm{Time}}_{t}+\beta_{3}{\left(\mathrm{Intervention}\times \mathrm{Time}\right)}_{jt}+\beta_{4}{T0}_{i}+\beta_{5}{\mathrm{Period}}_{k}+u_{i}+v_{il}+\varepsilon_{ijkt}$$

where *Y_ijkt_* is the plasma amino acid concentration for participant *i* under intervention *j* in period *k* (visit order) at time *t*, with $\beta_{0}$ as the intercept; $\beta_{1}$ and $\beta_{2}$ the main effects of intervention and time; $\beta_{3}$ the interaction term; $\beta_{4}$ the coefficient for the fasting concentration covariate; and $\beta_{5\;}$ the period effect. The terms $u_{i}$ and $v_{il}$ are random intercepts for participants and visit *l* within participants, accounting for between-person variability and correlation between repeated timepoints within each visit. $\varepsilon_{ijkt}$ is the residual error. Where the graphical inspection of residuals suggested violations of normality or non-constant variance, the outcome and T0 covariate were log-transformed (log[x + 1]) prior to refitting.

Analysis was run on both crude plasma concentrations (µmol·L^−1^), reflecting the absolute physiological response, and normalised with the TAA of the test biscuit consumed (µmol·L^−1^·g TAA^−1^) to enable the direct comparison of PPAR per unit of amino acid ingested.

#### 2.6.2. Analysis of Postprandial Kinetic Parameters

Postprandial kinetic parameters were derived non-parametrically from each individual baseline-corrected time–concentration profile using the trapezoidal rule on both raw and normalised datasets. The incremental area under curve (iAUC) was calculated over 0–120 min in two forms, net iAUC, which included the negative excursions below the fasting baseline to determine the overall postprandial response, and positive iAUC, with negative values set to zero to determine the raw postprandial increase after consumption. Peak postprandial free amino acid concentration above baseline (C_max_) was defined as the maximum observed baseline-corrected concentration across the postprandial sampling period. The time at which this peak was observed (T_max_) was also recorded. iAUC and C_max_ were calculated with both raw postprandial concentrations, expressed in units µmol·min·L^−1^ and µmol·L^−1^, respectively, and normalised to the TAA content of the biscuit consumed, expressed in units µmol·min·L^−1^·g TAA^−1^ and µmol·L^−1^·g TAA^−1^, respectively.

The effect of intervention on each postprandial kinetic parameter (iAUC, C_max_, T_max_) was evaluated separately for each amino acid using the LMM:

$$Y_{ijk}=\beta_{0}+\beta_{1}{\mathrm{Intervention}}_{j}+\beta_{2}{\mathrm{Period}}_{k}+u_{i}+\varepsilon_{ijk}$$

where $Y_{ijk}$ is the parameter value for participant *i* under intervention *j* in period (visit order) *k,* with $\beta_{0}$ as the intercept; $\beta_{1}$ and $\beta_{2}$ the fixed effects of intervention and period; $u_{i}$ the random intercept for a participant accounting for between-person variability and $\varepsilon_{ijk}$ the residual error. As a secondary approach to allow for an exploratory analysis of kinetic parameters with reduced dependence on the postprandial sampling schedule, kinetic parameters (AUC, C_max_, T_max_) were additionally estimated by fitting a Wood curve to each time–concentration series as per Wehrens et al. [28] and described in greater detail in Supplementary Method S1.

### 2.7. Power Calculation and Statistical Analysis

The assessment of PPAR was a secondary outcome of the parent clinical study, and it was hence not utilised for the determination of the original sample size estimates. A sensitivity analysis was conducted in G*Power version 3.1.9.7 (Heinrich-Heine-Universität Düsseldorf, Düsseldorf, Germany) as described by Faul et al. [29] to define the effect size that the completed study (*n* = 15) could detect. For a within-subject repeated-measures comparison across the three interventions (α = 0.05; power = 0.80; observed correlation among repeated measures = 0.53), this study was sensitive to only a medium to large effect (Cohen’s *f* = 0.33, as summarised in Serdar et al. [30]) for the iAUC of P-T21AA (µmol·min·L^−1^).

All statistical analyses were performed in R (version [4.4.3]; R Foundation for Statistical Computing, Vienna, Austria) using the lme4, lmerTest, and emmeans packages. Biscuit and flour amino acid composition data are reported as the mean ± standard deviation (SD). Group differences in the composition of three biscuits were analysed using a one-way analysis of variance (ANOVA) with group as the fixed factor, followed by pairwise comparisons using estimated marginal means with Bonferroni adjustment. Differences between the composition of the two flours were assessed using independent *t*-tests. The normality of residuals and homogeneity of variance were checked using the Shapiro–Wilk and Levene’s tests, respectively.

For graphical presentation purposes, PPAR time course parameters are reported as the baseline-corrected mean ± standard error of the mean (SEM), in both crude plasma concentrations (µmol·L^−1^), and further normalised with the TAA of the test biscuit consumed (µmol·L^−1^·g TAA^−1^). The main effects of intervention, time and their interaction were determined through omnibus Type III ANOVA F-tests, while timepoint significance was determined with Bonferroni-adjusted post hoc pairwise contrasts between interventions at each individual timepoint if the main interaction effects were significant. The results for the composite totals P-T21AA and P-EAA are considered the main outcomes of the analysis, while the time courses of individual amino acids are exploratory.

iAUC, T_max_, and C_max_ are expressed as the mean and 95% confidence intervals (CIs). Pairwise contrasts between all three intervention pairs were estimated using estimated marginal means with Bonferroni correction for the three simultaneous comparisons, with significance at a Bonferroni-adjusted *p* < 0.05. To control for multiplicity across all amino acids tested within each kinetic parameter, Benjamini–Hochberg (BH) false discovery rate (FDR) correction was applied, and amino acids that were significant after Bonferroni but not FDR correction were indicated but considered exploratory results. Figures were drawn using GraphPad Prism 9.3.0 software (GraphPad Software Inc., La Jolla, CA, USA).

## 3. Results

### 3.1. Amino Acid Profile in Powder and Biscuits

The TAA and FAA of AP-BSG and FP-BSG powders before incorporating them into biscuits can be found in Supplementary Tables S1 and S2 respectively. Although not significant, fermentation increased the TAA and total EAA (TEAA) overall in FP-BSG compared to AP-BSG. Besides methionine, phenylalanine, arginine, glutamic acid, proline and tyrosine, which were significantly reduced, and leucine, valine and threonine, which were not significantly increased, the other amino acids were found to have significantly increased.

Table 2 presents the TAA, TEAA and total non-essential amino acid (TNEAA) contents in all three biscuits. In general, ABSG and FBSG biscuits had significantly higher amounts of TAA, TEAA and several individual amino acids compared to Control biscuits, namely aspartic acid, threonine, serine, glycine, alanine, valine, leucine, tyrosine, phenylalanine, histidine and arginine. Additionally, FBSG biscuits further showed significantly higher isoleucine and lysine content compared to Control biscuits. Similarly, TNEAA was also significantly higher in ABSG and FBSG compared to Control biscuits, with no significant difference between BSG-containing biscuits. Proline, however, was significantly higher in ABSG only compared to Control biscuits, while cysteine was significantly lower in ABSG compared to FBSG.

Table 3 shows the FAA composition of the biscuits, expressed as total FAA (TF-AA), total free EAA (TF-EAA), total free NEAA (TF-NEAA), and total free twenty proteinogenic amino acids (TF-20AA), the standard twenty amino acids mentioned in Section 2.2.

Compared to ABSG and Control biscuits, FBSG biscuits showed significantly higher TF-AA, TF-EAA, and TF-20AA (Table 3). In addition, although minimal, some amount of non-proteinogenic FAA, or AAs not included in TF-20AA, was also detected only in FBSG biscuits.

### 3.2. AAS of Biscuits

Table 4 summarises the AAS for the three biscuits, and overall, the AASs of all indispensable amino acids, except lysine, exceeded the FAO reference pattern (AAS > 1.0), and ABSG and FBSG biscuits showed generally higher AASs than the Control biscuits. In particular, the AAS of lysine increased from 0.57 in Control biscuits to 0.61 in ABSG and 0.81 in FBSG biscuits, respectively.

### 3.3. Analysis of PPAR Time Course and Kinetic Parameters

Fifteen participants, ten males and five females, with a mean age of 63 ± 10 years, completed this study. All met at least three of the five MetS criteria, with six meeting four and one meeting all five. Detailed baseline characteristics of the recruited participants have been previously reported [26].

**Figure 2:**
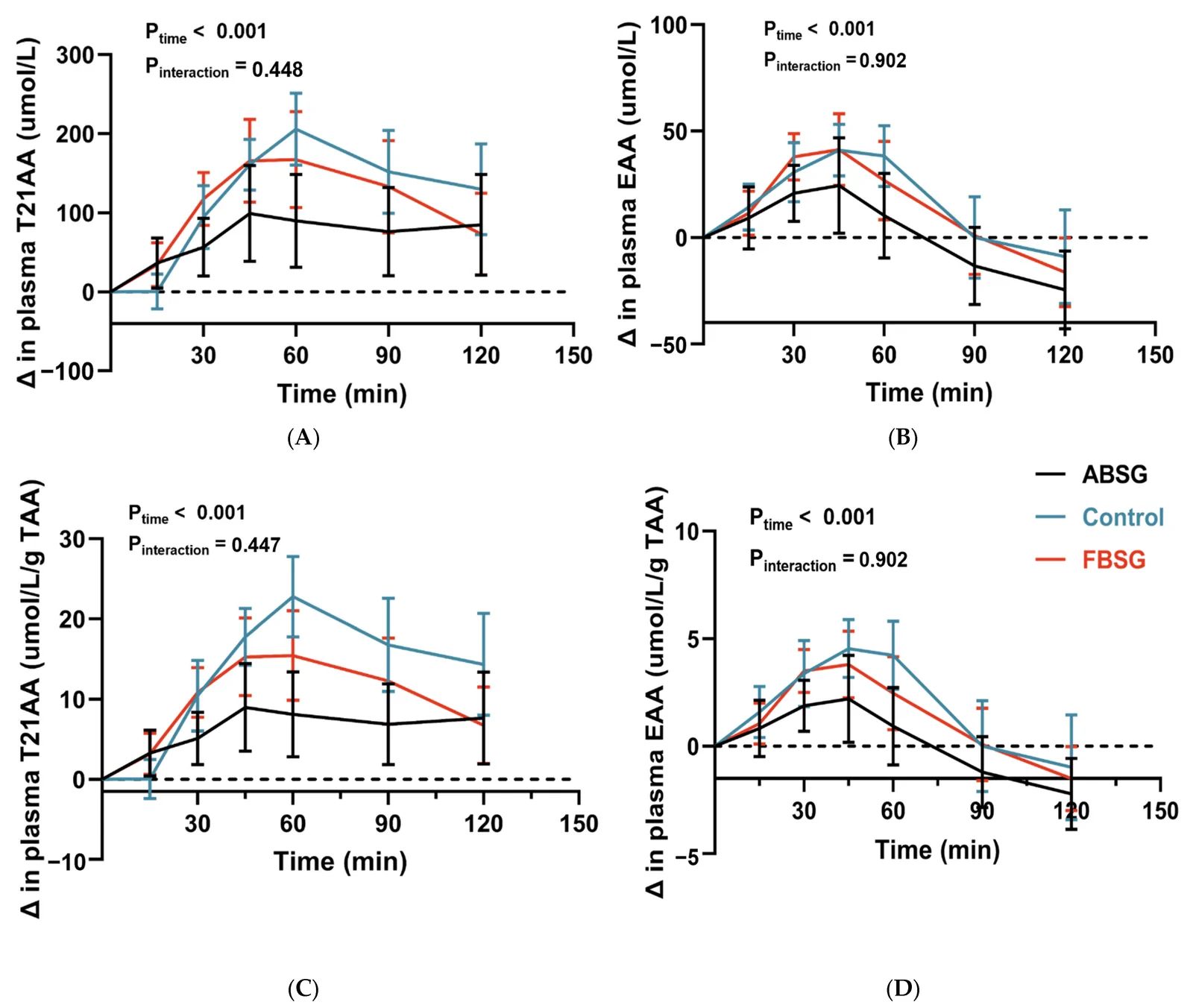
Changes in blood plasma concentration across 120 min. Absolute (raw) concentrations of (**A**) P-T21AA (µmol/L) and (**B**) P-EAA (µmol/L). Concentration normalised to TAA of biscuits in (**C**) P-T21AA/gram of TAA in biscuits and (**D**) P-EAA/gram of TAA in biscuits. All data are baseline-corrected, with the dashed line indicating zero change from baseline concentration. *p*-values were determined by linear mixed models with Bonferroni post hoc test. P-T21AA refers to (plasma) total 21 amino acids and P-EAA (plasma) essential amino acids, as defined in Section 2.6.

The PPARs for P-T21AA and P-EAA are shown in Figure 2, expressed in both raw concentrations (Figure 2A,B) and normalised to the TAA content of each biscuit (Figure 2C,D). For raw concentrations, significant time effects were noted for P-T21AA (*p* < 0.001) and P-EAA (*p* < 0.001), while there were no significant time x intervention interaction effects (P-T21AA: *p* = 0.448; P-EAA: *p* = 0.902). The normalised analysis (Figure 2C,D) showed similar results, with significant time but not interaction effects. The P-NEAA and individual amino acid PPAR raw concentration curves are shown in Supplementary Figures S1–S3, with significant interaction effects observed for cysteine only.

The net iAUCs of P-T21AA, P-EAA, P-NEAA and individual amino acids are represented in Table 5, while the net iAUC of other non-proteinogenic FAA in plasma, positive iAUC, T_max_ and C_max_ of all the amino acids in both raw and normalised concentrations are provided in Supplementary Tables S3–S6 and Supplementary Tables S7–S10 respectively.

When considering raw PPAR concentrations, no significant differences between groups were observed for any kinetic parameter of P-T21AA, P-EAA, or any individual amino acid (Supplementary Tables S3–S6). The only exception was the T_max_ of P-NEAA, which was significantly lower for FBSG compared to ABSG biscuits (Supplementary Table S5). Similarly, no significant differences were found for the normalised PPAR concentrations of P-T21AA and P-EAA (Supplementary Tables S7–S10). A small number of individual amino acids showed significant differences in kinetic parameters after normalisation, in particular glutamine, with a significantly higher net iAUC after the consumption of Control compared to ABSG, and proline, which had a significantly higher net and positive iAUC after the consumption of Control biscuits compared to both BSG biscuits (Supplementary Tables S7 and S8). However, it is important to note that none of the significant differences found in the kinetic parameter analysis remained significant after FDR correction.

When referring to the Wood curve analysis, model-derived parameters (Supplementary Tables S11–S13) were broadly similar to those of the traditional trapezoid estimates listed above, with no significant difference found for any kinetic parameters for P-T21AA or P-EAA. As only 61% of the Wood curves were successfully fitted (Supplementary Table S14), these results are considered more exploratory than confirmatory.

## 4. Discussion

Bakery products are widely consumed but are typically carbohydrate-dense foods with a limited nutrition profile, including low protein content and quality [31,32]. The incorporation of alternative protein-rich ingredients such as BSG into them has hence gained interest as a strategy to improve their nutritional profile and subsequently contribute to an overall sustainable and healthy eating pattern without needing to adopt drastic dietary changes [33,34]. In this study, the incorporation of BSG into biscuits significantly increased both TAA and FAA contents compared to Control biscuits, enriching the protein content. Fermentation was found to further improve the compositional aspect of protein quality in BSG, particularly by improving the AAS of lysine. However, despite improvements in the indispensable amino acid profile and protein content, this was not translated to significant improvements in the PPAR of amino acids in individuals with MetS.

Compared to Control biscuits, the addition of BSG increased TAA and EAA content in both ABSG and FBSG. Refined wheat flour is known to have low protein quality and insufficient amounts of amino acids such as lysine, methionine and threonine [35,36], and the increase in amino acid content observed here is consistent with the higher protein content in BSG [37]. Fermentation is generally reported to further increase protein and amino acid content through complementary routes. Mould contributes to protein content via the accumulation of fungal biomass and the excretion of proteinaceous extracellular enzymes [38,39]. The loss of non-starch polysaccharides degraded due to the metabolic activity of these enzymes also reduces total dry mass and hence increases relative protein content [40]. In addition, fungal proteases excreted during fermentation can break down storage proteins into smaller peptides and FAA, increasing soluble nitrogen fractions [41], which is consistent with the higher TF-AA content observed in FP-BSG compared to AP-BSG and consequently in FBSG compared to ABSG and Control biscuits. However, in the present study, TAA and EAA did not increase significantly at either powder or biscuit levels after fermentation. Increases in nitrogen content from fermentation with RO may be limited as it was reported to be incapable of nitrogen fixation and hence dependent on substrate nitrogen for fungal protein synthesis [42]. This contrasts with the literature reporting significant increases following BSG fermentation with *Rhizopus* spp. [41,43,44], which can be partially explained by differences in fermentation strain and conditions. Cooray and Chen [19] reported increases in amino acid content under comparable fermentation conditions, suggesting strain-dependent differences in dry mass loss, while differences from other studies may relate to time [41] and nitrogen supplementation [43].

Nevertheless, with respect to protein composition, fermentation resulted in a relatively more favourable amino acid profile in the biscuit as the fungus was shown to utilise certain amino acids, such as proline and glutamic acid during fungal growth [45,46], while showing a relative increase in certain amino acids, in particular lysine [44]. Sun et al. [47] further showed increased abundances of amino acid metabolites, particularly for essential amino acids including lysine, threonine and phenylalanine after the fermentation of a soybean meal with RO. In our study, the shift in composition was reflected in the AAS, in particular, for lysine, as fermentation increased the AAS of lysine to 0.81 in FBSG compared to 0.61 in ABSG and 0.57 in Control. Limiting amino acids and poor protein digestibility are the reasons for the low nutritional quality of plant proteins [48], and protein blends with complementary amino acid compositions blends are therefore commonly used to increase the AAS of fortified foods [49,50], although single substitutions can be effective [51]. Hoehnel et al. [52] used a combination of three high-protein ingredients of buckwheat, faba bean and lupin to increase the AAS of pasta from 0.57 to 0.87, with lysine as the limiting amino acid. The comparable improvement in the lysine AAS in our biscuits shows that fermentation can be a potential single-ingredient alternative to blending that can also improve the AAS. In addition, fermentation also increased the non-proteinogenic amino acids present in both FP-BSG and FBSG, including γ-Aminobutyric acid, which may be metabolic byproducts of fungal growth [46,53]. The physiological implications of their increased concentrations in the diet remain unclear, as many non-proteinogenic amino acids serve primarily as intermediates in metabolic pathways [54]. β-alanine, for example, acts as a substrate for carnosine synthesis, which can act as an antioxidant and delay muscle fatigue [55]; however, the present study was not designed to evaluate such downstream metabolic effects.

Beyond amino acid composition, fermentation has been generally shown to improve protein digestibility, largely attributed to how the protein structure is modified during microbial metabolism to increase solubility or further broken down to smaller peptides through the enzymatic activity of the enzymes released during the process of fermentation [56,57]. A reduction in anti-nutritional factors such as fibre and phytates also improves protein digestibility [58]. In addition, fungal protein may also be more digestible compared to plant protein, as fungal cell walls are known to be more porous, dynamic and flexible compared to plant cell walls, which could increase the level of protein hydrolysis during digestion [59]. Using a modified INFOGEST digestion model, Wang et al. [60] showed that edible filamentous fungi from RO had similar levels of the degree of hydrolysis and amino acid accessibility to salmon, chicken and beef muscle proteins, although this could differ from the cultivation environment of the fungal biomass [61]. Taken together, these mechanisms suggest that RO fermentation could theoretically improve the digestibility and nutritional quality of BSG protein. Nevertheless, in vitro digestion models are generally considered simplified approximations of in vivo processes and cannot fully reproduce the physiological conditions of human digestion [62,63], which the present study assessed by measuring PPAR in vivo.

Despite the theoretical benefits of fermentation, no significant differences were observed in the majority of the PPAR curves, time to peak, or iAUC between interventions. This is consistent with a recent systematic review and meta-analysis, which found that processing-induced improvements in in vitro protein digestibility were not consistently reflected in in vivo postprandial amino acid responses [64]. Differences in absolute protein dose may partly explain this discrepancy. In contrast with the present findings, Perez-Moral et al. [65] reported a higher PPAR following the consumption of chickpea cell-enriched breads compared to regular white bread, reflective of the higher EAA content present. Their enriched breads provided 17–26 g of protein per intervention, compared to the 9–11 g of protein per intervention in this study. Similarly, Lee et al. [66] observed no significant differences in PPAR following the consumption of fermented okara in biscuits compared to regular wheat biscuits with a protein content of approximately 7–8 g. Collectively, these findings suggest that relatively modest protein doses may be insufficient to elicit detectable differences in systemic amino acid appearances.

The structural properties of the BSG matrix may have also limited protein accessibility during digestion. Although not significantly different, PPARs for both BSG interventions appeared to be lower than that for Control, especially for ABSG despite containing higher protein content. This may be due to whole BSG having a poorly accessible matrix that is rich in fibre and lignocellulosic cell wall material derived from barley husk and endosperm fractions. These structural components can encapsulate proteins and limit enzymatic accessibility during gastrointestinal digestion, resulting in slower protein hydrolysis and delayed gastric emptying, which may further attenuate early PPAR [67]. In comparison, while fermentation may be able to partially modify the BSG structure, Canedo, de Paula, da Silva and Vendruscolo [43] noted that RO spores were able to immobilise the surface of BSG for growth without disrupting its structure, which could limit the extent to which protein accessibility is improved. In addition, a BSG protein isolate as a plant protein may also have lower sensitivity to protein hydrolysis due to its crosslinking and folding [68].

Several physiological factors may also contribute to the null difference in PPAR, though these mechanisms could not be directly assessed in the present study and are proposed as hypothetical explanations. First-pass splanchnic extraction has been reported to increase with age [69,70]. Given the older age range of the participants in this study (mean age of 63 years), a greater proportion of absorbed amino acids may have undergone hepatic uptake or oxidation without substantially altering systemic appearance. In addition, the metabolic characteristics of the study population may have further reduced sensitivity in detecting between-intervention differences. Yanni et al. [71] observed a lower plasma iAUC response to the same plant protein-enriched biscuit in overweight versus normal-weight adults, indicating that metabolic impairments may attenuate PPAR. In individuals with MetS, metabolic alterations such as insulin resistance and elevated levels of fasting branched-chain and aromatic amino acids may mask any relatively small between-intervention differences [70,72,73].

The strengths of this study include the comprehensive assessment of protein composition and the resulting PPAR after the consumption of BSG-containing foods, rather than supplementation in an extracted form, allowing for amino acid kinetics to be evaluated in a realistic mixed meal bakery matrix. Furthermore, this study was conducted on the MetS population, which may particularly benefit from dietary strategies aimed at improving protein quality, as higher dietary protein quality has been reported to be protective against MetS-related risk factors [74]. Nevertheless, there are some existing limitations. Firstly, although BSG was incorporated at a relatively high substitution level, the protein dose delivered within a standard biscuit serving remained modest, which may have limited the magnitude of postprandial responses, as previously discussed. Delivering a higher protein dose would require greater substitution beyond levels generally compatible with acceptable sensory and physical properties [75]. In addition, PPAR was a secondary outcome, and this study had limited statistical power to detect smaller between-intervention differences. Furthermore, a longer postprandial duration may be required to capture the full amino acid response. Plant proteins tend to produce smaller, delayed increases in PPAR [76], with PPAR being further delayed in older populations [77]. Certain amino acids, such as branched-chain amino acids, were also shown to display a secondary rise 6–8 h after a meal [78]. Lastly, the absence of a healthy control group limits the ability to determine whether the observed amino acid responses were influenced primarily by the BSG interventions or by metabolic characteristics specific to individuals with MetS.

## 5. Conclusions

To conclude, these findings suggest that fermentation can improve the amino acid composition of BSG, particularly by increasing the amino acid score of lysine and free amino acid content present. However, at the realistic substitution dosage studied, these compositional improvements did not result in detectable differences in systemic amino acid exposure during the early 0–120 min postprandial period in individuals with MetS. While the fermentation of BSG remains a promising means of upcycling it into a more nutritionally favourable ingredient, future studies incorporating interventions with complementary amino acid composition blending, longer postprandial sampling periods, or population groups with different health statuses can provide a more comprehensive understanding of its effects on PPAR.

## Figures and Tables

**Table 1:** Nutritional composition of biscuits and flours.

Nutritional Composition	Biscuits (/90 g Dry Biscuit)			Flours (/100 g)		
	Control	ABSG	FBSG	Wheat Flour ^#^	AP-BSG	FP-BSG
Protein (g) *	9.2 ± 0.6	10.6 ± 0.8	10.9 ± 0.7	11.0	21.8 ± 0.5	25.3 ± 0.7
Fat (g)	19.3 ± 1.2	20.4 ± 1.5	19.9 ± 1.3	1.20	9.9 ± 0.2	7.8 ± 0.2
ACHO (g)	62.6 ± 4.1	50.2 ± 3.8	47.9 ± 3.1	68.2	6.6 ± 0.2	4.0 ± 0.1
TDF (g)	2.0 ± 0.26	10.9 ± 0.8	10.6 ± 0.6	2.9	58.4 ± 1.3	57.3 ± 1.7
IDF (g)	1.3 ± 0.2	10.1 ± 0.8	9.5 ± 0.6	-	56.7 ± 1.3	54.3 ± 1.7
SDF (g)	0.7 ± 0.1	0.8 ± 0.1	1.1 ± 0.1	-	1.7 ± 0.0	3.0 ± 0.2
Phytic Acid (g)	0.7 ± 0.0	1.0 ± 0.1	0.9 ± 0.1	-	2.4 ± 0.1	2.1 ± 0.1

ABSG: autoclaved BSG biscuit; FBSG: fermented BSG biscuit; AP-BSG: autoclaved BSG powder; FP-BSG: fermented BSG powder; ACHO: available carbohydrate; TDF: total dietary fibre; IDF: insoluble dietary fibre; SDF: soluble dietary fibre. * Proteins of biscuits and flours were calculated with protein conversion factor of 6.25. ^#^ Nutritional information was derived from commercial packaging (Prima Plain Flour, Singapore). Values are from [24].

**Table 2:** Content of amino acids from hydrolysis in each of three biscuits.

Amino Acids (mg)	Concentration of Amino Acid (/100 g Dry Biscuit)		
	Control	ABSG	FBSG
Histidine	209.3 ± 4.1 ^a^	253.8 ± 7.9 ^b^	261.8 ± 10.5 ^b^
Isoleucine	359.1 ± 32.3 ^a^	449.3 ± 42.5 ^ab^	477.1 ± 45.7 ^b^
Leucine	673.5 ± 13.2 ^a^	864.1 ± 40.3 ^b^	827.5 ± 24.8 ^b^
Lysine	264.7 ± 3.9 ^a^	325.3 ± 50.0 ^a^	440.9 ± 28.5 ^b^
Methionine	282.9 ± 39.5 ^a^	278.0 ± 17.9 ^a^	273.2 ± 17.0 ^a^
Phenylalanine	480.4 ± 10.9 ^a^	623.3 ± 33.7 ^b^	570.7 ± 16.0 ^b^
Threonine	361.1 ± 11.5 ^a^	481.4 ± 27.6 ^b^	485.5 ± 21.3 ^b^
Tryptophan	100.3 ± 1.6 ^a^	116.6 ± 21.4 ^a^	116.3 ± 2.2 ^a^
Valine	407.3 ± 34.4 ^a^	518.3 ± 28.6 ^b^	521.0 ± 53.0 ^b^
TEAA	3138.4 ± 45.2 ^a^	3910.2 ± 200.3 ^b^	3974.0 ± 157.8 ^b^
Alanine	327.7 ± 8.5 ^a^	458.1 ± 20.3 ^b^	470.4 ± 17.5 ^b^
Arginine	373.9 ± 4.6 ^a^	524.0 ± 29.6 ^b^	513.3 ± 17.7 ^b^
Aspartic acid	534.1 ± 6.0 ^a^	752.0 ± 30.0 ^b^	789.1 ± 25.5 ^b^
Cysteine	134.3 ± 4.9 ^ab^	80.3 ± 16.3 ^a^	211.9 ± 53.1 ^b^
Glutamic acid	2738.3 ± 110.6 ^a^	2910.7 ± 131.9 ^a^	2615.2 ± 88.7 ^a^
Glycine	293.8 ± 6.2 ^a^	381.2 ± 17.6 ^b^	381.8 ± 10.4 ^b^
Proline	847.6 ± 28.8 ^a^	1006.9 ± 52.1 ^b^	840.0 ± 21.2 ^a^
Serine	480.4 ± 12.5 ^a^	584.5 ± 22.8 ^b^	562.9 ± 21.1 ^b^
Tyrosine	350.5 ± 12.1 ^a^	459.7 ± 24.1 ^b^	475.1 ± 12.1 ^b^
TNEAA	6080.4 ± 159.8 ^a^	7157.2 ± 298.8 ^b^	6859.6 ± 235.7 ^b^
TAA	9218.8 ± 174.5 ^a^	11,067.5 ± 479.3 ^b^	10,833.6 ± 369.0 ^b^

ABSG: autoclaved BSG biscuit; FBSG: fermented BSG biscuit. TEAA: total essential amino acids; TNEAA: total non-essential amino acids; TAA: total amino acids. Values are presented as mean ± SD, *n* = 3. Different alphabet superscripts indicate significant differences across all groups, *p* ≤ 0.05 via one-way ANOVA with Bonferroni post hoc test. Asparagine and glutamine are not quantified as they are converted to aspartic acid and glutamic acid during acid hydrolysis.

**Table 3:** Content of FAA per gram of defatted biscuit.

Free Amino Acids (µmol)	Concentration of Amino Acid (/g Defatted Biscuit)		
	Control	ABSG	FBSG
Histidine	0.03 ± 0.01 ^a^	0.07 ± 0.02 ^a^	1.26 ± 0.18 ^b^
Isoleucine	0.10 ± 0.02 ^a^	0.16 ± 0.03 ^a^	0.92 ± 0.08 ^b^
Leucine	0.13 ± 0.03 ^a^	0.22 ± 0.07 ^a^	1.16 ± 0.18 ^b^
Lysine	0.07 ± 0.02 ^a^	0.16 ± 0.03 ^a^	1.57 ± 0.25 ^b^
Methionine	0.03 ± 0.01 ^a^	0.08 ± 0.04 ^a^	0.32 ± 0.05 ^b^
Phenylalanine	0.07 ± 0.01 ^a^	0.24 ± 0.01 ^a^	0.89 ± 0.13 ^b^
Threonine	0.12 ± 0.02 ^a^	0.17 ± 0.04 ^a^	1.52 ± 0.14 ^b^
Tryptophan	0.14 ± 0.06 ^a^	0.14 ± 0.06 ^a^	0.31 ± 0.07 ^b^
Valine	0.20 ± 0.01 ^a^	0.25 ± 0.05 ^a^	1.46 ± 0.14 ^b^
TF-EAA	0.90 ± 0.18 ^a^	1.50 ± 0.09 ^a^	9.42 ± 1.17 ^b^
Alanine	0.38 ± 0.07 ^a^	0.44 ± 0.09 ^a^	4.06 ± 0.32 ^b^
Arginine	0.14 ± 0.03 ^a^	0.23 ± 0.02 ^a^	0.75 ± 0.09 ^b^
Asparagine	0.52 ± 0.10 ^a^	0.39 ± 0.03 ^a^	1.82 ± 0.21 ^b^
Aspartic acid	1.01 ± 0.20 ^a^	0.68 ± 0.15 ^a^	1.83 ± 0.19 ^b^
Cysteine	0.01 ± 0.00 ^a^	0.01 ± 0.01 ^a^	0.20 ± 0.03 ^b^
Glutamic acid	0.59 ± 0.17 ^a^	0.51 ± 0.14 ^a^	1.98 ± 0.18 ^b^
Glutamine	ND	0.04 ± 0.07 ^a^	2.05 ± 0.57 ^b^
Glycine	0.17 ± 0.02 ^a^	0.17 ± 0.03 ^a^	1.91 ± 0.18 ^b^
Proline	0.16 ± 0.06 ^a^	0.52 ± 0.09 ^b^	1.34 ± 0.12 ^c^
Serine	0.18 ± 0.04 ^a^	0.23 ± 0.01 ^a^	1.77 ± 0.17 ^b^
Tyrosine	0.08 ± 0.02 ^a^	0.14 ± 0.02 ^a^	1.02 ± 0.14 ^b^
TF-NEAA	3.24 ± 0.72 ^a^	3.35 ± 0.50 ^a^	18.72 ± 1.96 ^b^
TF-20AA	4.14 ± 0.89 ^a^	4.85 ± 0.54 ^a^	28.14 ± 3.11 ^b^
α-aminoadipic (α-AAA)	ND	ND	0.24 ± 0.10 ^b^
β-Alanine	0.02 ± 0.01 ^a^	0.04 ± 0.02 ^a^	0.71 ± 0.02 ^b^
Citrulline	ND	ND	0.06 ± 0.01 ^b^
Cystathionine (Cysthi)	ND	ND	0.11 ± 0.03 ^b^
Ethanolamine (EOHNH_2_)	0.09 ± 0.06 ^a^	0.06 ± 0.02 ^a^	0.88 ± 0.12 ^b^
γ-amino-n-butyric acid (g-ABA)	0.08 ± 0.02 ^a^	0.14 ± 0.01 ^a^	1.64 ± 0.15 ^b^
Ornithine	ND	0.01 ± 0.01 ^a^	0.50 ± 0.10 ^b^
O-phosphoethanolamine (PEA)	0.05 ± 0.02 ^a^	0.32 ± 0.08 ^b^	0.64 ± 0.04 ^c^
O-phospho-L-Serine (P-Ser)	0.16 ± 0.03 ^a^	0.14 ± 0.03 ^a^	0.80 ± 0.03 ^b^
Sarcosine	ND	ND	0.24 ± 0.03 ^b^
Taurine	0.14 ± 0.00 ^a^	0.35 ± 0.17 ^ab^	0.45 ± 0.03 ^b^
TF-AA	4.67 ± 0.89 ^a^	5.92 ± 0.54 ^a^	34.41 ± 3.56 ^b^

ABSG: autoclaved BSG biscuit; FBSG: fermented BSG biscuit; ND: no peak detected; TF-EAA: total free essential amino acids; TF-NEAA: total free non-essential amino acids; TF-20AA: total free twenty proteinogenic amino acids; TF-AA: total free amino acids. Data expressed as mean ± SD; *n* = 3. Different alphabet superscripts indicate significant differences across all groups, *p* ≤ 0.05 via one-way ANOVA with Bonferroni post hoc test.

**Table 4:** Amino acid score of three biscuits.

Essential Amino Acids	Mean Concentration of Amino Acid (mg/g Protein)				AAS		
	FAO ^#^	Control	ABSG	FBSG	Control	ABSG	FBSG
Histidine	15.00	20.42	21.51	21.69	1.36	1.43	1.45
Isoleucine	30.00	35.03	38.08	39.53	1.17	1.27	1.32
Leucine	59.00	65.70	73.23	68.56	1.11	1.24	1.16
Lysine	45.00	25.82	27.57	36.53	0.57	0.61	0.81
Methionine + Cysteine	22.00	40.70	30.37	40.19	1.85	1.38	1.83
Phenylalanine + Tyrosine	38.00	81.06	91.78	86.65	2.13	2.42	2.28
Threonine	23.00	35.23	40.79	40.22	1.53	1.77	1.75
Tryptophan	6.00	9.78	9.88	9.64	1.63	1.65	1.61
Valine	39.00	39.73	43.93	43.17	1.02	1.13	1.11

ABSG: autoclaved BSG biscuit; FBSG: fermented BSG biscuit; AAS: amino acid score. ^#^ FAO adult reference patterns were derived from FAO/WHO/UNU adult reference pattern [25]. Values of protein and amino acid reported for each biscuit were average of triplicate measurements as reported in Table 1 (re-expressed to 100 g) and Table 2 respectively.

**Table 5:** Net iAUC of amino acids in plasma.

Amino Acid	iAUC (µmol·min · L^−1^)		
	Control	ABSG	FBSG
Histidine	228.3 (−23.0, 479.6)	183.7 (−98.8, 466.1)	226.6 (−36.5, 489.7)
Isoleucine	320.5 (−169.3, 810.3)	22.7 (−425.8, 471.1)	174.7 (−253.4, 602.8)
Leucine	353.0 (−479.2, 1185.2)	−124.5 (−918.1, 669.1)	137.1 (−573.7, 847.9)
Lysine	−383.5 (−849.8, 82.8)	−478.5 (−1355.3, 398.2)	−17.1 (−654.6, 620.4)
Methionine	42.2 (−87.5, 172.0)	−33.3 (−159.8, 93.2)	40.8 (−78.1, 159.7)
Phenylalanine	538.9 (259.3, 818.4)	386.5 (62.2, 710.9)	427.0 (166.0, 688.0)
Threonine	572.3 (286.5, 858.2)	278.8 (−131.6, 689.3)	584.3 (144.3, 1024.2)
Tryptophan	91.3 (−59.8, 242.3)	123.2 (−26.1, 272.5)	43.2 (−105.1, 191.5)
Valine	283.6 (−460.4, 1027.7)	−151.9 (−957.8, 653.9)	284.6 (−396.8, 965.9)
P-EAA	2046.7 (−1169.3, 5262.6)	206.6 (−3616.5, 4029.8)	1901.1 (−1407.9, 5210.1)
Alanine	6423.7 (4096.0, 8751.4)	4694.5 (1727.9, 7661.2)	5779.2 (3117.7, 8440.7)
Arginine	−20.7 (−441.5, 400.1)	−1.6 (−452.4, 449.1)	262.8 (−130.7, 656.2)
Asparagine	226.2 (18.6, 433.9)	193.0 (−31.5, 417.5)	258.6 (53.3, 463.9)
Aspartic acid	−17.4 (−60.4, 25.6)	−48.9 (−89.4, −8.4)	−48.9 (−86.6, −11.3)
Cysteine	−387.4 (−756.2, −18.7)	−169.2 (−382.6, 44.2)	−37.7 (−279.4, 204.0)
Glutamine	2083.2 (156.9, 4009.5)	−136.0 (−2498.2, 2226.1)	1529.3 (−813.5, 3872.1)
Glutamic acid	−974.6 (−1703.4, −245.7)	−613.8 (−1337.7, 110.1)	−1059.3 (−1719.0, −399.5)
Glycine	368.3 (−155.3, 891.8)	362.2 (−252.5, 977.0)	262.3 (−424.5, 949.0)
Proline	5247.6 (4019.3, 6475.8)	4353.5 (3252.2, 5454.9)	4289.6 (3004.0, 5575.1)
Serine	876.2 (451.9, 1300.5)	478.6 (32.5, 924.6)	814.6 (333.2, 1296.0)
Taurine	−524.4 (−1063.9, 15.2)	−687.5 (−1118.6, −256.4)	−359.3 (−823.0, 104.4)
Tyrosine	−7.7 (−208.7, 193.4)	−152.0 (−415.2, 111.1)	−40.5 (−277.7, 196.6)
P-NEAA	13,293.1 (7530.0, 19,056.2)	8272.8 (438.6, 16,107.0)	11,650.5 (4362.4, 18,938.7)
P-T21AA	15,339.7 (6805.3, 23,874.2)	8479.4 (−2647.1, 19,605.9)	13,551.6 (3061.2, 24,042.1)

ABSG: autoclaved BSG biscuit; FBSG: fermented BSG biscuit; P-EAA: (plasma) essential amino acids; P-NEAA: (plasma) non-essential amino acids; P-T21AA: (plasma) total 21 amino acids. These are all as defined in Section 2.6. Net iAUC was calculated from t = 0 to t = 120 min using trapezoidal rule on baseline-corrected data. Values are represented as mean (95% CI). Pairwise comparisons between interventions were performed using linear mixed models with Bonferroni correction. No significant differences were found.

## Data Availability

The data presented in this study will be made available upon request to JEK (fstkje@nus.edu.sg).

## Supplementary Materials

The following supporting information can be downloaded at https://www.mdpi.com/article/10.3390/foods15193558/s1, Supplementary Method S1: Wood curve fitting; Supplementary Table S1: Content of amino acids from hydrolysis in AP-BSG and FP-BSG; Supplementary Table S2: Content of FAA in AP-BSG and FP-BSG; Supplementary Table S3: Table of net iAUC for non-proteinogenic amino acids detected in plasma; Supplementary Table S4: Table of positive iAUC of amino acids in plasma; Supplementary Table S5: Table of peak time (T_max_) for raw plasma amino acid concentrations; Supplementary Table S6: Table of highest peak concentration (C_max_) for raw plasma amino acid concentrations; Supplementary Table S7: Table of net iAUC of amino acids in plasma per gram of TAA in biscuits; Supplementary Table S8: Table of positive iAUC of amino acids in plasma per gram of TAA in biscuits; Supplementary Table S9: Table of peak time (T_max_) for normalised plasma amino acid concentrations; Supplementary Table S10: Table of highest peak concentration (C_max_) for normalised plasma amino acid concentrations; Supplementary Table S11: Table of AUC from curated Wood curves and imputation; Supplementary Table S12: Table of peak time (T_max_) from curated Wood fits; Supplementary Table S13: Table of highest peak concentration (C_max_) from curated Wood curves and imputation; Supplementary Table S14: Summary of curated Wood curves’ accepted rates; Supplementary Figure S1: Change in non-proteinogenic AA in blood plasma; Supplementary Figure S2: Change in EAA in blood plasma; Supplementary Figure S3: Change in NEAA in blood plasma.

## Abbreviations

The following abbreviations are used in this manuscript:

AAS	Amino Acid Score
ABSG	Autoclaved Brewer’s Spent Grain Biscuit
ANCOVA	Analysis Of Covariance
ANOVA	Analysis Of Variance
AP-BSG	Autoclaved Brewer’s Spent Grain Powder
AUC	Area Under The Curve
BSG	Brewer’s Spent Grain
BH	Benjamini–Hochberg
CI	Confidence Interval
C_max_	Peak Postprandial Concentration Above Baseline
EAAs	Essential Amino Acids
FAAs	Free Amino Acids
FAO	Food and Agriculture Organization of the United Nations
FBSG	Fermented Brewer’s Spent Grain Biscuit
FDR	False Discovery Rate
FP-BSG	Fermented Brewer’s Spent Grain Powder
iAUC	Incremental Area Under The Curve
LMM	Linear Mixed Model
MetS	Metabolic Syndrome
NCEP-ATP III	National Cholesterol Education Program Adult Treatment Panel III
NEAAs	Non-Essential Amino Acids
P-EAA	Plasma Essential Amino Acids
P-NEAA	Plasma Non-Essential Amino Acids
PPAR	Postprandial Plasma Amino Acid Response
P-T21AA	Plasma Total 21 Amino Acids (20 Proteinogenic Amino Acids + Taurine)
RO	*Rhizopus oligosporus*
SD	Standard Deviation
SEM	Standard Error Of The Mean
TAAs	Total Amino Acids
TCA	Trichloroacetic Acid
TEAAs	Total Essential Amino Acids
TF-AA	Total Free Amino Acids
TF-EAA	Total Free Essential Amino Acids
TF-NEAA	Total Free Non-Essential Amino Acids
TF-20AA	Total Free Twenty Proteinogenic Amino Acids
T_max_	Time To Peak Concentration
TNEAAs	Total Non-Essential Amino Acids
