# Supplementary material for: Protein Composition and Postprandial Aminoacidemia of Biscuits Fortified with Brewer’s Spent Grain and Fermented Brewer’s Spent Grain in Individuals with Metabolic Syndrome
Source: Foods. 2026 Oct 7;15(19):3558. doi: 10.3390/foods15193558 (PMC13664953; doi:10.3390/foods15193558)
Supplement: Supplementary file 1 [file foods-15-03558-s001.zip › foods-4564900-supplementary.pdf]

Supplementary Materials

Protein composition and postprandial aminoacidemia of biscuits fortified with brewer's spent grain and fermented brewer's spent grain in individuals with metabolic syndrome

Zi Ning Leong[1], Yujing Xu[1], Jialin Chen[1], Yidan Chang[1], Gregory Chung Tsing Chan[2], Jung Eun Kim[1,3] *

[1] Department of Food Science and Technology, Faculty of Science, National University of Singapore, Singapore

[2] Saw Swee Hock School of Public Health, National University of Singapore, Singapore

[3] Bezos Center for Sustainable Protein, National University of Singapore, Singapore

* Correspondence: Jung Eun Kim, fstkje@nus.edu.sg

List of Supplementary Methods

Supplementary Method S1: Wood curve fitting 6

List of Supplementary Tables

Supplementary Table S1: Content of amino acids from hydrolysis in AP-BSG and FP-BSG 8

Supplementary Table S2: Content of FAA in AP-BSG and FP-BSG 9

Supplementary Table S3: Table of net iAUC for non-proteinogenic amino acids detected in plasma 11

Supplementary Table S4: Table of positive iAUC of amino acids in plasma 12

Supplementary Table S5: Table of peak time (T_{max}) for raw plasma amino acid concentrations 14

Supplementary Table S6: Table of highest peak concentration (C_{max}) for raw plasma amino acid concentrations 15

Supplementary Table S7: Table of net iAUC of amino acids in plasma per gram of TAA in biscuits 16

Supplementary Table S8: Table of positive iAUC of amino acids in plasma per gram of TAA in biscuits 18

Supplementary Table S9: Table of peak time (T_{max}) for normalised plasma amino acids concentrations 20

Supplementary Table S10: Table of highest peak concentration (C_{max}) for normalised plasma amino acid concentrations 21

Supplementary Table S11: Table of AUC from curated Wood curves and imputation 22

Supplementary Table S12: Table of peak time (T_{max}) from curated Wood fits 24

Supplementary Table S13: Table of highest peak concentration (C_{max}) from curated Wood curves and imputation 25

Supplementary Table S14: Summary of curated Wood curves accepted rates..................... 27

List of Supplementary Figures

Supplementary Figure S1: Change in non-proteinogenic AA in blood plasma. 28

Supplementary Figure S2: Change in EAA in blood plasma 31

Supplementary Figure S3: Change in NEAA in blood plasma. 35

Supplementary Method S1: Wood curve fitting

In order to account for outlying observations in postprandial amino-acid time courses, kinetic parameters were additionally estimated by fitting a Wood curve to each individual amino-acid time-concentration series (0-120 min) using the R package aaresponse as described by Wehrens et al. [28] as a secondary analysis to the primary trapezoidal estimation. This approach allows for continuous kinetic parameter estimation independent of the sampling schedule, while modelling the characteristic rise-and-fall trajectory of the postprandial response. As the baseline concentration for Wood curves are estimated as part of the curve-fitting procedure, area under the curve (AUC) rather than incremental AUC (iAUC) was computed, peak concentration (C_{max}) and time to peak (T_{max}) were defined as in the primary analysis. Plasma amino acids used for the Wood-curve fitting were normalised to TAA content of the biscuit consumed.

After fitting, visual curation was performed to ensure all fits were physiologically plausible and consistent with the raw data. Curves were rejected if they were (1) monotonically declining with no discernible rise, (2) flat with no response, or (3) poorly fitted to the observed data (e.g. V-shaped or biphasic profiles that the unimodal Wood function could not capture). Exceptions were made for amino acids that did not reach a peak within the observation window, as these indicated a potential absorptive response despite lacking a clear rise-and-fall trajectory. These curves were capped at time and concentration = 120 minutes for all reporting purposes.

After curation, kinetic parameters were extracted from all accepted fits using the extractParameters function of the aaresponse package. For participant–intervention–amino-acid time courses with no acceptable Wood fit, missing AUC and C_{max} values were imputed by robust linear regression. For each amino acid, regression models were fitted across participants relating the kinetic parameter to the inter-quantile range (80th–20th percentile) of observed raw values, and missing values were predicted from each participant's raw postprandial concentrations. A summary of the curated fits acceptance rates can be found in **Supplementary Table S14**, with a total of 61% of curves accepted. Non-proteinogenic amino acids were found to be the least likely to follow the fitted Wood Curves.

The effect of intervention on each Wood-derived parameter (AUC, C_{max}, T_{max}) was evaluated separately for each amino acid using the same linear mixed model as the primary analysis. Amino acid concentrations normalised to the total amino acids of the biscuits were used for this analysis. Pairwise contrasts between all three intervention pairs were estimated

using estimated marginal means with Bonferroni correction for the three simultaneous comparisons, with significance at Bonferroni-adjusted $p < 0.05$. As the Wood curve fitting was exploratory for this study, further global multiplicity tests such as Benjamini-Hochberg False Discovery Rate applied in the primary analysis were not considered.

Supplementary Table S1: Content of amino acids from hydrolysis in AP-BSG and FP-BSG

Amino acids (mg)	Concentration of amino acid (/100g dry powder)		P-value
	AP-BSG	FP-BSG	
Histidine	389.64±11.03[a]	420.69±10.68[b]	0.025
Isoleucine	727.28±18.50[a]	1452.84±364.98[b]	0.026
Leucine	1300.32±50.62[a]	1347.49±112.45[a]	0.544
Lysine	728.83±19.41[a]	932.45±48.81[b]	0.003
Methionine	463.10±55.27[a]	100.69±71.60[b]	0.002
Phenylalanine	946.20±86.90[a]	675.06±56.53[b]	0.011
Threonine	807.33±57.02[a]	970.92±86.68[a]	0.052
Tryptophan	120.75±3.60[a]	375.17±1.39[b]	0.000
Valine	975.92±50.16[a]	1069.28±31.15[a]	0.052
TEAA	6459.38±129.75[a]	7344.57±569.14[a]	0.058
Alanine	891.20±107.43[a]	1673.12±172.21[b]	0.003
Arginine	903.81±14.32[a]	635.09±19.17[b]	0.000
Aspartic acid	1191.41±16.01[a]	1553.08±41.61[b]	0.000
Cysteine	248.45±18.59[a]	434.80±17.57[b]	0.000
Glutamic acid	3300.79±217.01[a]	2194.91±76.42[b]	0.001
Glycine	675.76±33.28[a]	1399.96±72.44[b]	0.000
Proline	1599.66±157.99[a]	1073.58±27.78[b]	0.005
Serine	686.52±55.82[a]	953.26±77.39[b]	0.008
Tyrosine	660.32±58.24[a]	524.25±57.97[b]	0.046
TAA	16617.30±729.49[a]	17786.62±949.19[a]	0.166

AP-BSG: Autoclaved BSG flour; FP-BSG: Fermented BSG flour; TEAA: Total essential amino acids; TAA: Total amino acids. Values are expressed as mean ± SD; n = 3. Different lower-case letters indicate significant differences between AP-BSG and FP-BSG within each amino acid, $p \leq 0.05$, based on estimated marginal means with Bonferroni adjustment. Asparagine and glutamine are not quantified as they are converted to aspartic acid and glutamic acid during acid hydrolysis.

Supplementary Table S2: Content of FAA in AP-BSG and FP-BSG

Free Amino acids (µmol)	Concentration of amino acid (/g dry powder)	
	**AP-BSG**	**FP-BSG**
Histidine	0.15±0.01[a]	5.62±0.41[b]
Isoleucine	0.19±0.01[a]	2.59±0.27[b]
Leucine	0.48±0.02[a]	4.13±0.37[b]
Lysine	0.29±0.01[a]	5.45±0.38[b]
Methionine	0.05±0.00[a]	1.54±0.19[b]
Phenylalanine	0.39±0.02[a]	3.26±0.46[b]
Threonine	0.17±0.01[a]	5.48±0.34[b]
Tryptophan	0.12±0.01[a]	1.03±0.26[b]
Valine	0.44±0.02[a]	4.31±0.38[b]
TF-EAA	2.29±0.11[a]	33.41±2.90[b]
Alanine	0.54±0.02[a]	13.79±1.05[b]
Arginine	0.43±0.02[a]	2.18±0.17[b]
Asparagine	0.18±0.01[a]	5.03±0.39[b]
Aspartic acid	0.18±0.01[a]	3.70±0.29[b]
Cysteine	ND	0.78±0.10[b]
Glutamic acid	0.39±0.02[a]	5.50±0.49[b]
Glutamine	0.24±0.01[a]	19.85±1.59[b]
Glycine	0.17±0.01[a]	6.57±0.48[b]
Proline	1.54±0.07[a]	4.21±0.37[b]
Serine	0.20±0.01[a]	5.81±0.47[b]
Tyrosine	0.26±0.01[a]	4.02±0.39[b]
TF-NEAA	4.13±0.19[a]	71.42±5.66[b]
TF-20AA	6.43±0.30[a]	104.84±8.48[b]
1-methyl-L-histidine (1Mehis)	ND	0.11±0.02[b]
α -aminoadipic (α-AAA)	ND	1.96±0.14[b]
α -aminobutyric acid (α-ABA)	ND	0.60±0.11[b]
Anserine	ND	0.39±0.03[b]
β-Aminoisobutyric acid (β-AiBA)	0.05±0.00[a]	0.86±0.30[b]
β-Alanine	0.05±0.01[a]	2.01±1.01[b]
Citrulline	ND	0.23±0.06[b]
Cystathionine (Cysthi)	ND	0.26±0.08[b]
Ethanolamine ($EOHNH_2$)	0.11±0.01[a]	3.19±0.33[b]
γ-amino-n-butyric acid (g-ABA)	0.25±0.01[a]	6.26±0.60[b]
Hydroxylysine (Hylys)	ND	0.07±0.02[b]
Ornithine	0.05±0.00[a]	2.74±0.17[b]
O-phosphoethanolamine (PEA)	0.28±0.01[a]	0.89±0.02[b]
O-phospho-L-Serine (P-Ser)	0.15±0.01[a]	1.45±0.13[b]
Sarcosine	ND	1.25±0.09[b]
Taurine	0.23±0.01[a]	0.95±0.05[b]
TF-AA	7.61±0.35[a]	128.05±11.39[b]

AP-BSG: Autoclaved BSG flour; FP-BSG: Fermented BSG flour, ND: No peak detected; TF-EAA: Total free essential amino acids; TF-NEAA: Total free non-essential amino acids; TF-20AA; Total free twenty proteinogenic amino acids; TFAA: Total free amino acids. Data are expressed as mean ± SD; n = 3. Different

lower-case letters indicate significant differences between AP-BSG and FP-BSG within each amino acid, $p \leq 0.05$, based on estimated marginal means with Bonferroni adjustment.

Supplementary Table S3: Table of net iAUC for non-proteinogenic amino acids detected in plasma

Amino Acid	Net iAUC (µmol·min ·L^{-1})		
	Control	ABSG	FBSG
α-ABA	68.01 (-14.67, 150.68)	68.60 (-26.13, 163.34)	65.32 (-39.46, 170.11)
Cit	-266.97 (-425.92, -108.02)	-100.81 (-268.84, 67.21)	-90.83 (-265.50, 83.85)
Orn	459.27 (14.25, 904.28)	236.86 (-125.38, 599.09)	228.27 (-195.44, 651.98)
PEA	-132.78 (-356.80, 91.24)	-137.35 (-453.14, 178.43)	-217.64 (-444.91, 9.64)
P-SER	-159.66 (-449.25, 129.93)	-102.46 (-429.63, 224.71)	-328.32 (-583.45, -73.19)

α-ABA: α -aminobutyric acid; Cit: Citrulline; Orn: Ornithine; PEA: O-phosphoethanolamine; P-Ser: O-phospho-L-Serine ND: No peak detected. Net iAUC was calculated from t = 0 to t = 120 min using the trapezoidal rule on baseline-corrected data. Values are represented as mean (95% CI). Pairwise comparisons between interventions were performed using linear mixed models with Bonferroni correction. No significant differences were observed.

Supplementary Table S4: Table of positive iAUC of amino acids in plasma

Amino Acid	Positive iAUC (µmol·min ·L^{-1})		
	Control	ABSG	FBSG
P-T21AA	17266.99 (9585.70, 24948.28)	13738.83 (5429.13, 22048.52)	16206.48 (7285.44, 25127.52)
P-EAA	3811.48 (1274.29, 6348.66)	3306.88 (902.75, 5711.00)	3865.09 (1365.58, 6364.61)
P-NEAA	14312.22 (8905.95, 19718.50)	10864.85 (4503.68, 17226.02)	13067.27 (6773.20, 19361.35)
α-ABA	110.14 (57.34, 162.94)	129.47 (83.28, 175.65)	134.06 (55.78, 212.34)
Ala	6669.53 (4417.88, 8921.18)	5332.83 (2723.54, 7942.12)	6330.61 (4201.12, 8460.10)
Arg	321.80 (157.79, 485.80)	352.44 (87.86, 617.02)	471.45 (187.13, 755.78)
Asn	339.53 (182.43, 496.62)	281.27 (101.27, 461.26)	346.89 (164.06, 529.73)
Asp	28.20 (0.26, 56.14)	17.36 (2.87, 31.85)	9.95 (3.43, 16.46)
Cit	32.66 (0.60, 64.71)	81.93 (24.41, 139.45)	109.37 (2.42, 216.32)
Cys	69.81 (20.19, 119.44)	99.10 (12.14, 186.06)	179.14 (57.07, 301.21)
Gln	2970.37 (1552.72, 4388.03)	1915.21 (597.97, 3232.44)	2833.94 (1273.41, 4394.46)
Glu	276.14 (22.66, 529.62)	399.84 (-50.75, 850.44)	202.84 (68.40, 337.27)
Gly	722.06 (378.83, 1065.29)	725.74 (307.97, 1143.51)	644.56 (104.63, 1184.49)
His	336.38 (154.35, 518.40)	317.52 (109.06, 525.98)	329.72 (115.29, 544.16)
Ile	529.48 (118.28, 940.69)	405.37 (164.61, 646.14)	446.88 (146.82, 746.94)
Leu	837.33 (179.73, 1494.93)	645.46 (235.50, 1055.42)	685.80 (213.94, 1157.66)
Lys	308.08 (127.94, 488.21)	443.81 (-99.31, 986.93)	561.35 (182.43, 940.27)
Met	129.38 (44.82–213.93)	91.94 (23.46–160.42)	118.01 (31.90–204.12)
Orn	579.56 (173.14, 985.99)	430.31 (172.12, 688.50)	448.42 (177.91, 718.93)
PEA	168.91 (29.92, 307.89)	217.86 (59.35, 376.38)	111.54 (44.57, 178.51)
Phe	587.02 (335.27, 838.76)	465.01 (191.00, 739.02)	492.81 (280.35, 705.27)
Pro	5258.14 (4036.99, 6479.29)	4373.36 (3286.48, 5460.24)	4355.85 (3120.58, 5591.13)
P-SER	191.31 (4.64, 377.98)	201.57 (21.20, 381.95)	97.09 (-3.45, 197.63)
Ser	951.33 (570.86, 1331.80)	655.09 (300.98, 1009.20)	929.76 (523.11, 1336.42)
Tau	228.58 (-6.93, 464.08)	113.78 (38.12, 189.44)	228.19 (34.54, 421.83)
Thr	662.14 (421.99, 902.29)	508.72 (207.74, 809.71)	733.13 (350.26, 1116.00)
Trp	180.86 (71.94, 289.79)	194.01 (85.75, 302.28)	149.27 (83.69, 214.86)
Tyr	193.59 (89.19, 298.00)	162.45 (25.38, 299.53)	196.53 (80.85, 312.21)
Val	730.08 (117.32, 1342.83)	621.83 (165.83, 1077.82)	735.62 (232.36, 1238.88)

P-EAA: (Plasma) essential amino acids; P-NEAA: (Plasma) non-essential amino acids; P-T21AA: (Plasma) total amino acids, all as defined in Section 2.6. Abbreviations of amino acids were used in the table. Positive iAUC was calculated from t = 0 to t = 120 min using the positive trapezoidal rule on baseline-corrected data. Values are represented as mean (95% CI) in µmol·min·L^{-1}. Pairwise comparisons between interventions were performed using linear mixed models with Bonferroni correction. No significant differences were observed.

Supplementary Table S5: Table of peak time (T_{max}) for raw plasma amino acid concentrations

Amino Acid	T_{max} (min) for raw plasma amino acid concentrations					
	Control		ABSG		FBSG	
	n	Average (95% CI)	n	Average (95% CI)	n	Average (95% CI)
P-T21AA	15	67.00 (49.84, 84.16)	12	75.00 (48.66, 101.34)	15	60.00 (44.30, 75.70)
P-EAA	14	46.07 (28.93, 63.21)	12	56.25 (35.48, 77.02)	14	51.43 (35.60, 67.25)
P-NEAA	**15**	**83.00 (66.73, 99.27)[ab]**	**13**	**87.69 (66.81, 108.58)[a]**	**15**	**63.00 (45.75, 80.25)[b]**
α-ABA	14	60.00 (37.21, 82.79)	15	57.00 (42.21, 71.79)	14	58.93 (39.57, 78.28)
Ala	15	93.00 (76.93, 109.07)	15	89.00 (71.67, 106.33)	14	77.14 (64.05, 90.24)
Arg	12	76.25 (52.74, 99.76)	11	79.09 (54.37, 103.81)	13	61.15 (40.73, 81.58)
Asn	14	63.21 (42.50, 83.93)	14	54.64 (33.54, 75.75)	15	59.00 (38.54, 79.46)
Asp	9	60.00 (27.39, 92.61)	11	60.00 (31.50, 88.50)	9	50.00 (17.39, 82.61)
Cit	7	85.71 (44.32, 127.11)	12	72.50 (47.50, 97.50)	10	88.50 (59.69, 117.31)
Cys	10	49.50 (26.85, 72.15)	10	45.00 (20.74, 69.26)	13	38.08 (20.09, 56.06)
Gln	13	79.62 (60.25, 98.98)	11	68.18 (38.16, 98.20)	13	49.62 (32.91, 66.32)
Glu	10	30.00 (14.82, 45.18)	11	35.45 (19.04, 51.87)	10	40.50 (17.85, 63.15)
Gly	12	80.00 (57.99, 102.01)	12	82.50 (56.32, 108.68)	14	55.71 (34.66, 76.77)
His	14	63.21 (49.13, 77.29)	11	81.82 (60.03, 103.60)	14	55.71 (37.29, 74.14)
Ile	13	51.92 (35.12, 68.73)	12	48.75 (34.03, 63.47)	14	51.43 (35.60, 67.26)
Leu	13	50.77 (34.00, 67.54)	12	48.75 (34.03, 63.47)	14	51.43 (35.60, 67.26)
Lys	13	36.92 (23.27, 50.58)	9	53.33 (22.23, 84.44)	13	35.77 (22.67, 48.87)
Met	12	67.50 (44.33, 90.67)	9	75.00 (45.04, 104.96)	14	53.57 (38.11, 69.03)
Orn	15	59.00 (41.96, 76.04)	15	62.00 (45.13, 78.87)	15	77.00 (54.83, 99.17)
P-SER	9	63.33 (21.85, 104.82)	12	56.25 (31.16, 81.34)	9	50 (18.95, 81.05)
PEA	13	55.38 (34.65, 76.12)	11	55.91 (29.60, 82.22)	13	65.77 (38.95, 92.59)
Phe	15	69.00 (51.30, 86.70)	14	79.29 (61.17, 97.40)	15	58.00 (42.99, 73.01)
Pro	15	102.00 (88.24, 115.76)	15	94.00 (76.67, 111.33)	15	94.00 (76.67, 111.33)
Ser	15	49.00 (33.47, 64.53)	14	52.50 (33.06, 71.94)	14	49.29 (32.16, 66.42)
Tau	10	34.50 (10.21, 58.79)	9	38.33 (2.74, 73.92)	12	40.00 (21.23, 58.77)
Thr	15	50.00 (30.73, 69.27)	13	60.00 (37.49, 82.51)	15	50.00 (37.98, 62.02)
Trp	13	54.23 (37.46, 71.00)	14	50.36 (31.87, 68.84)	13	43.85 (26.70, 60.99)
Tyr	13	55.38 (39.52, 71.25)	9	51.67 (29.26, 74.08)	13	49.62 (33.75, 65.48)
Val	14	51.43 (34.89, 67.97)	12	46.25 (28.32, 64.18)	14	51.43 (35.60, 67.26)

T_{max}: Time to peak concentration. P-EAA: (Plasma) essential amino acids; P-NEAA: (Plasma) non-essential amino acids; P-T21AA: (Plasma) total amino acids, all as defined in Section 2.6. Abbreviations of amino acids were used in the table. Data is expressed as mean (95% CI) in minutes. Participants with missing peaks for individual amino acids were omitted. Pairwise comparisons between interventions were performed using linear mixed models with Bonferroni correction. Only amino acids with at least one significant pairwise difference between interventions were annotated and bolded, with values not sharing a common superscript letter differing significantly at $P \leq 0.05$. No difference remained significant after BH-FDR correction.

Supplementary Table S6: Table of highest peak concentration (C_{max}) for raw plasma amino acid concentrations

Amino Acid	C_{max} (µmol·L⁻¹) for raw plasma amino acid concentrations					
	Control		ABSG		FBSG	
	n	Average (95% CI)	n	Average (95% CI)	n	Average (95% CI)
P-T21AA	15	264.37 (167.95, 360.78)	12	279.81 (154.33, 405.30)	15	256.06 (156.61, 355.50)
P-EAA	14	69.80 (41.06, 98.55)	12	71.20 (28.35, 114.06)	14	73.24 (45.06, 101.41)
P-NEAA	15	208.98 (137.17, 280.80)	13	205.63 (109.87, 301.40)	15	195.43 (122.13, 268.73)
α-ABA	14	2.13 (1.29, 2.97)	15	2.99 (1.59, 4.39)	14	2.49 (1.56, 3.42)
Ala	15	97.72 (65.20, 130.24)	15	86.64 (46.38, 126.91)	14	98.52 (71.74, 125.30)
Arg	12	7.68 (5.14, 10.23)	11	8.79 (4.02, 13.55)	13	8.93 (5.26, 12.61)
Asn	14	5.81 (3.83, 7.79)	14	4.86 (2.12, 7.59)	15	5.91 (3.65, 8.17)
Asp	9	0.89 (0.39, 1.39)	11	0.74 (0.32, 1.15)	9	0.48 (0.27, 0.70)
Cit	7	2.42 (0.70, 4.15)	12	2.64 (1.42, 3.85)	10	3.65 (0.93, 6.37)
Cys	10	2.16 (1.38, 2.93)	10	3.61 (0.21, 7.02)	13	4.62 (2.27, 6.96)
Gln	13	60.53 (37.71, 83.35)	11	52.20 (22.69, 81.70)	13	55.47 (33.02, 77.91)
Glu	10	9.72 (4.55, 14.89)	11	15.08 (6.24, 23.91)	10	8.79 (5.32, 12.26)
Gly	12	17.30 (11.44, 23.15)	12	17.18 (9.43, 24.93)	14	12.27 (4.59, 19.96)
His	14	5.46 (2.96, 7.97)	11	6.89 (3.74, 10.05)	14	5.62 (3.14, 8.09)
Ile	13	9.90 (4.81, 15.00)	12	9.15 (5.25, 13.06)	14	8.18 (4.83, 11.53)
Leu	13	16.35 (8.46, 24.23)	12	14.80 (7.62, 21.97)	14	13.40 (7.90, 18.90)
Lys	13	8.08 (5.21, 10.95)	9	13.72 (-0.73, 28.17)	13	12.41 (7.24, 17.59)
Met	12	2.64 (1.48, 3.80)	9	2.62 (1.31, 3.94)	14	2.19 (1.12, 3.25)
Orn	15	9.15 (4.51, 13.79)	15	8.08 (3.94, 12.22)	15	7.39 (4.30, 10.48)
P-SER	9	10.22 (0.49, 19.96)	12	5.46 (1.96, 8.96)	9	4.44 (2.09, 6.79)
PEA	13	4.89 (1.41, 8.37)	11	7.51 (3.01, 12.01)	13	4.70 (2.52, 6.88)
Phe	15	8.31 (5.56, 11.07)	14	7.44 (3.97, 10.90)	15	7.48 (4.93, 10.03)
Pro	15	69.25 (53.62, 84.87)	15	61.22 (46.52, 75.92)	15	60.63 (46.42, 74.83)
Ser	15	14.84 (10.19, 19.49)	14	11.75 (7.25, 16.25)	14	15.32 (10.94, 19.70)
Tau	10	6.88 (2.66, 11.11)	9	8.11 (3.93, 12.29)	12	7.68 (4.64, 10.71)
Thr	15	11.69 (7.96, 15.42)	13	9.99 (5.60, 14.39)	15	11.95 (7.80, 16.10)
Trp	13	3.41 (2.16, 4.66)	14	3.67 (1.92, 5.42)	13	3.37 (1.96, 4.78)
Tyr	13	4.46 (3.21, 5.72)	9	5.31 (2.15, 8.47)	13	4.46 (2.74, 6.17)
Val	14	15.44 (6.98, 23.90)	12	14.82 (5.57, 24.07)	14	14.63 (8.82, 20.44)

C_{max}: Concentration at highest peak. P-EAA: (Plasma) essential amino acids; P-NEAA: (Plasma) non-essential amino acids; P-T21AA: (Plasma) total amino acids, all as defined in Section 2.6. Abbreviations of amino acids were used in the table. Data is expressed as mean (95% CI) in µmol·L⁻¹. Participants with missing peaks for individual amino acids were omitted. Pairwise comparisons between interventions were performed using linear mixed models with Bonferroni correction. No significant differences were observed.

Supplementary Table S7: Table of net iAUC of amino acids in plasma per gram of TAA in biscuits

Amino Acid	Net iAUC (µmol·min ·L^{-1} ·g TAA^{-1})		
	**Control**	**ABSG**	**FBSG**
P-T21AA	1663.96 (738.20, 2589.73)	766.19 (-239.19, 1771.57)	1250.96 (282.58, 2219.34)
P-EAA	222.01 (-126.84, 570.85)	18.67 (-326.78, 364.12)	175.49 (-129.97, 480.95)
P-NEAA	1441.96 (816.81, 2067.10)	747.52 (39.63, 1455.41)	1075.47 (402.69, 1748.24)
α-ABA	7.38 (-1.59, 16.35)	6.20 (-2.36, 14.76)	6.03 (-3.64, 15.70)
Ala	696.81 (444.31, 949.30)	424.19 (156.13, 692.25)	533.48 (287.80, 779.17)
Arg	-2.25 (-47.89, 43.40)	-0.15 (-40.87, 40.58)	24.26 (-12.06, 60.58)
Asn	24.54 (2.02, 47.06)	17.44 (-2.84, 37.73)	23.87 (4.92, 42.82)
Asp	-1.89 (-6.55, 2.78)	-4.42 (-8.08, -0.76)	-4.52 (-7.99, -1.04)
Cit	-28.96 (-46.20, -11.72)	-9.11 (-24.29, 6.07)	-8.38 (-24.51, 7.74)
Cys	-42.03 (-82.02, -2.03)	-15.29 (-34.57, 3.99)	-3.48 (-25.80, 18.83)
Gln	**225.98 (17.02, 434.93)[a]**	**-12.29 (-225.73, 201.15)[b]**	**141.17 (-75.09, 357.43)[ab]**
Glu	-105.71 (-184.77, -26.65)	-55.46 (-120.87, 9.95)	-97.78 (-158.68, -36.88)
Gly	39.95 (-16.85, 96.74)	32.73 (-22.82, 88.28)	24.21 (-39.18, 87.61)
His	24.77 (-2.49, 52.03)	16.60 (-8.93, 42.12)	20.92 (-3.37, 45.21)
Ile	34.77 (-18.36, 87.89)	2.05 (-38.47, 42.57)	16.12 (-23.40, 55.64)
Leu	38.29 (-51.98, 128.56)	-11.25 (-82.96, 60.46)	12.66 (-52.96, 78.27)
Lys	-41.60 (-92.18, 8.98)	-43.24 (-122.46, 35.98)	-1.58 (-60.43, 57.26)
Met	4.58 (-9.49, 18.66)	-3.01 (-14.44, 8.42)	3.77 (-7.21, 14.74)
Orn	49.82 (1.55, 98.09)	21.40 (-11.33, 54.13)	21.07 (-18.04, 60.18)
PEA	-14.40 (-38.70, 9.90)	-12.41 (-40.94, 16.12)	-20.09 (-41.07, 0.89)
Phe	58.45 (28.13, 88.77)	34.93 (5.62, 64.24)	39.42 (15.33, 63.51)
Pro	**569.22 (435.99, 702.46)[a]**	**393.38 (293.86, 492.89)[b]**	**395.97 (277.30, 514.64)[b]**
P-SER	-17.32 (-48.73, 14.09)	-9.26 (-38.82, 20.30)	-30.31 (-53.86, -6.76)
Ser	95.05 (49.02, 141.07)	43.24 (2.93, 83.55)	75.20 (30.76, 119.63)
Tau	-56.88 (-115.41, 1.64)	-62.12 (-101.07, -23.17)	-33.17 (-75.97, 9.63)
Thr	62.08 (31.08, 93.09)	25.20 (-11.89, 62.28)	53.93 (13.32, 94.54)
Trp	9.90 (-6.48, 26.28)	11.13 (-2.36, 24.62)	3.99 (-9.70, 17.68)
Tyr	-0.83 (-22.64, 20.98)	-13.74 (-37.51, 10.04)	-3.74 (-25.63, 18.15)
Val	30.77 (-49.94, 111.48)	-13.73 (-86.54, 59.08)	26.27 (-36.63, 89.16)

P-EAA: (Plasma) essential amino acids; P-NEAA: (Plasma) non-essential amino acids; P-T21AA: (Plasma) total amino acids, all as defined in Section 2.6. Abbreviations of amino acids were used in the table. Net iAUC

was calculated from t = 0 to t = 120 min using the trapezoidal rule on baseline-corrected data. Values are represented as mean (95% CI) in $\mu mol \cdot min \cdot L^{-1} \cdot g\ TAA^{-1}$. Pairwise comparisons between interventions were performed using linear mixed models with Bonferroni correction. Only amino acids with at least one significant pairwise difference between interventions were annotated and bolded, with values not sharing a common superscript letter differing significantly at $P \leq 0.05$. No difference remained significant after BH-FDR correction.

Supplementary Table S8: Table of positive iAUC of amino acids in plasma per gram of TAA in biscuits

Amino Acid	Positive iAUC (μmol·min ·L^{-1} ·g TAA^{-1})		
	Control	ABSG	FBSG
P-T21AA	1873.02 (1039.80, 2706.24)	1241.42 (490.57, 1992.28)	1496.03 (672.52, 2319.53)
P-EAA	413.45 (138.23, 688.66)	298.81 (81.57, 516.04)	356.79 (126.06, 587.52)
P-NEAA	1552.50 (966.06, 2138.94)	981.73 (406.95, 1556.52)	1206.25 (625.24, 1787.26)
α-ABA	11.95 (6.22, 17.68)	11.70 (7.52, 15.87)	12.38 (5.15, 19.6)
Ala	723.47 (479.22, 967.72)	481.87 (246.1, 717.64)	584.38 (387.81, 780.96)
Arg	34.91 (17.12, 52.70)	31.85 (7.94, 55.75)	43.52 (17.27, 69.77)
Asn	36.83 (19.79, 53.87)	25.42 (9.15, 41.68)	32.02 (15.14, 48.90)
Asp	3.06 (0.03, 6.09)	1.57 (0.26, 2.88)	0.92 (0.32, 1.52)
Cit	3.54 (0.06, 7.02)	7.4 (2.20, 12.6)	10.09 (0.22, 19.97)
Cys	7.57 (2.19, 12.96)	8.95 (1.10, 16.81)	16.54 (5.27, 27.8)
Gln	322.21 (168.43, 475.99)	173.05 (54.03, 292.08)	261.6 (117.55, 405.66)
Glu	29.95 (2.46, 57.45)	36.13 (-4.59, 76.85)	18.72 (6.31, 31.13)
Gly	78.32 (41.09, 115.56)	65.58 (27.83, 103.33)	59.5 (9.66, 109.34)
His	36.49 (16.74, 56.23)	28.69 (9.86, 47.53)	30.44 (10.64, 50.23)
Ile	57.44 (12.83, 102.04)	36.63 (14.87, 58.38)	41.25 (13.55, 68.95)
Leu	90.83 (19.50, 162.16)	58.32 (21.28, 95.37)	63.31 (19.75, 106.86)
Lys	33.42 (13.88, 52.96)	40.1 (-8.97, 89.18)	51.82 (16.84, 86.8)
Met	14.03 (4.86, 23.21)	8.31 (2.12, 14.5)	10.89 (2.94, 18.84)
Orn	62.87 (18.78, 106.95)	38.88 (15.55, 62.21)	41.39 (16.42, 66.36)
PEA	18.32 (3.25, 33.40)	19.69 (5.36, 34.01)	10.3 (4.11, 16.48)
Phe	63.68 (36.37, 90.98)	42.02 (17.26, 66.78)	45.49 (25.88, 65.1)
Pro	**570.37 (437.91, 702.83)[a]**	**395.17 (296.96, 493.38)[b]**	**402.09 (288.06, 516.12)[b]**
P-SER	20.75 (0.50, 41.00)	18.21 (1.91, 34.51)	8.96 (-0.32, 18.24)
Ser	103.19 (61.92, 144.47)	59.19 (27.2, 91.19)	85.83 (48.29, 123.37)
Tau	24.79 (-0.75, 50.34)	10.28 (3.44, 17.12)	21.06 (3.19, 38.94)
Thr	71.83 (45.78, 97.87)	45.97 (18.77, 73.16)	67.68 (32.33, 103.02)
Trp	19.62 (7.80, 31.43)	17.53 (7.75, 27.31)	13.78 (7.72, 19.84)
Tyr	21.00 (9.67, 32.32)	14.68 (2.29, 27.06)	18.14 (7.46, 28.82)
Val	79.19 (12.73, 145.66)	56.19 (14.98, 97.39)	67.91 (21.45, 114.36)

P-EAA: (Plasma) essential amino acids; P-NEAA: (Plasma) non-essential amino acids; P-T21AA: (Plasma) total amino acids, all as defined in Section 2.6. Abbreviations of amino acids were used in the table. Positive

iAUC was calculated from t = 0 to t = 120 min using the positive trapezoidal rule on baseline-corrected data. Values are represented as mean (95% CI) in $\mu mol \cdot min \cdot L^{-1} \cdot g\ TAA^{-1}$. Pairwise comparisons between interventions were performed using linear mixed models with Bonferroni correction. Only amino acids with at least one significant pairwise difference between interventions were annotated and bolded, with values not sharing a common superscript letter differing significantly at $P \leq 0.05$. No difference remained significant after BH-FDR correction.

Supplementary Table S9: Table of peak time (T_{max}) for normalised plasma amino acids concentrations

Amino Acid	T_{max} (min) for normalised plasma concentration					
	Control		ABSG		FBSG	
	n	Average (95% CI)	n	Average (95% CI)	n	Average (95% CI)
P-T21AA	15	67 (49.84, 84.16)	12	75.00 (48.66, 101.34)	15	60.00 (44.30, 75.70)
P-EAA	14	46.07 (28.93, 63.21)	12	56.25 (35.48, 77.02)	14	51.43 (35.60, 67.26)
P-NEAA	**15**	**83 (66.73, 99.27)[ab]**	**13**	**87.69 (66.81, 108.58)[a]**	**15**	**63 (45.75, 80.25)[b]**
α-ABA	14	61.07 (38.68, 83.47)	15	57.00 (42.21, 71.79)	14	58.93 (39.57, 78.28)
Ala	15	93.00 (76.93, 109.07)	15	89.00 (71.67, 106.33)	14	77.14 (64.05, 90.24)
Arg	12	76.25 (52.74, 99.76)	11	79.09 (54.37, 103.81)	13	61.15 (40.73, 81.58)
Asn	14	63.21 (42.50, 83.93)	14	54.64 (33.54, 75.75)	15	59 (38.54, 79.46)
Asp	9	60 (27.39, 92.61)	11	60 (31.50, 88.50)	11	50 (17.39, 82.61)
Cit	7	85.71 (44.32, 127.11)	12	72.5 (47.50, 97.50)	9	95 (66.76, 123.24)
Cys	10	49.5 (26.85, 72.15)	10	45 (20.74, 69.26)	13	38.08 (20.09, 56.06)
Gln	13	79.62 (60.25, 98.98)	11	68.18 (38.16, 98.2)	13	49.62 (32.91, 66.32)
Glu	10	30 (14.82, 45.18)	11	35.45 (19.04, 51.87)	10	40.5 (17.85, 63.15)
Gly	12	80 (57.99, 102.01)	12	82.5 (56.32, 108.68)	14	55.71 (34.66, 76.77)
His	14	63.21 (49.13, 77.29)	11	81.82 (60.03, 103.6)	14	55.71 (37.29, 74.14)
Ile	13	51.92 (35.12, 68.73)	12	48.75 (34.03, 63.47)	14	51.43 (35.60, 67.26)
Leu	13	50.77 (34.00, 67.54)	12	48.75 (34.03, 63.47)	14	51.43 (35.60, 67.26)
Lys	13	36.92 (23.27, 50.58)	9	53.33 (22.23, 84.44)	13	35.77 (22.67, 48.87)
Met	12	67.5 (44.33, 90.67)	9	75 (45.04, 104.96)	14	53.57 (38.11, 69.03)
Orn	15	59 (41.96, 76.04)	15	62 (45.13, 78.87)	15	77 (54.83, 99.17)
P-SER	9	63.33 (21.85, 104.82)	12	56.25 (31.16, 81.34)	9	50 (18.95, 81.05)
PEA	13	55.38 (34.65, 76.12)	11	55.91 (29.6, 82.22)	13	65.77 (38.95, 92.59)
Phe	15	69 (51.30, 86.7)	14	79.29 (61.17, 97.4)	15	58 (42.99, 73.01)
Pro	15	102 (88.24, 115.76)	15	94 (76.67, 111.33)	15	94 (76.67, 111.33)
Ser	15	49 (33.47, 64.53)	14	52.5 (33.06, 71.94)	14	49.29 (32.16, 66.42)
Tau	10	34.5 (10.21, 58.79)	9	38.33 (2.74, 73.92)	12	40 (21.23, 58.77)
Thr	15	50 (30.73, 69.27)	13	60 (37.49, 82.51)	15	50 (37.98, 62.02)
Trp	13	54.23 (37.46, 71)	14	50.36 (31.87, 68.84)	13	43.85 (26.7, 60.99)
Tyr	13	55.38 (39.52, 71.25)	9	51.67 (29.26, 74.08)	13	49.62 (33.75, 65.48)
Val	14	51.43 (34.89, 67.97)	12	46.25 (28.32, 64.18)	13	50.77 (33.6, 67.94)

T_{max}: Time to peak concentration. P-EAA: (Plasma) essential amino acids; P-NEAA: (Plasma) non-essential amino acids; P-T21AA: (Plasma) total amino acids, all as defined in Section 2.6. Abbreviations of amino acids were used in the table. Data is expressed as mean (95% CI) in minutes. Participants with missing peaks for individual amino acids were omitted. Pairwise comparisons between interventions were performed using linear mixed models with Bonferroni correction. Only amino acids with at least one significant pairwise difference between interventions were annotated and bolded, with values not sharing a common superscript letter differing significantly at $P \leq 0.05$. No difference remained significant after BH-FDR correction.

Supplementary Table S10: Table of highest peak concentration (C_{max}) for normalised plasma amino acid concentrations

Amino Acid	C_{max} (µmol·L^{-1}·g TAA^{-1})					
	**Control**		**ABSG**		**FBSG**	
	n	Average (95% CI)	n	Average (95% CI)	n	Average (95% CI)
P-T21AA	15	28.68 (18.22, 39.14)	12	25.28 (13.94, 36.62)	15	23.64 (14.46, 32.82)
P-EAA	14	7.57 (4.45, 10.69)	12	6.43 (2.56, 10.31)	14	6.76 (4.16, 9.36)
P-NEAA	15	22.67 (14.88, 30.46)	13	18.58 (9.93, 27.23)	15	18.04 (11.27, 24.81)
α-ABA	14	0.23 (0.14, 0.32)	15	0.27 (0.14, 0.4)	14	0.23 (0.14, 0.32)
Ala	15	10.60 (7.07, 14.13)	15	7.83 (4.19, 11.47)	14	9.09 (6.62, 11.57)
Arg	12	0.83 (0.56, 1.11)	11	0.79 (0.36, 1.22)	13	0.83 (0.49, 1.16)
Asn	14	0.63 (0.42, 0.84)	14	0.44 (0.19, 0.69)	15	0.55 (0.34, 0.75)
Asp	**9**	**0.10 (0.04, 0.15)[a]**	**11**	**0.07 (0.03, 0.10)[ab]**	**9**	**0.04 (0.02, 0.06)[b]**
Cit	7	0.26 (0.08, 0.45)	12	0.24 (0.13, 0.35)	9	0.37 (0.1, 0.64)
Cys	10	0.23 (0.15, 0.32)	10	0.32 (0.02, 0.63)	13	0.42 (0.21, 0.64)
Gln	13	6.57 (4.09, 9.04)	11	4.72 (2.05, 7.38)	13	5.12 (3.05, 7.19)
Glu	10	1.05 (0.49, 1.61)	11	1.36 (0.57, 2.16)	10	0.81 (0.49, 1.13)
Gly	12	1.88 (1.24, 2.51)	12	1.55 (0.85, 2.25)	14	1.13 (0.42, 1.84)
His	14	0.59 (0.32, 0.86)	11	0.62 (0.34, 0.91)	14	0.52 (0.29, 0.75)
Ile	13	1.07 (0.52, 1.63)	12	0.83 (0.47, 1.18)	14	0.76 (0.45, 1.06)
Leu	13	1.77 (0.92, 2.63)	12	1.34 (0.69, 1.99)	14	1.23 (0.73, 1.74)
Lys	13	0.88 (0.56, 1.19)	9	1.24 (-0.07, 2.54)	13	1.15 (0.67, 1.62)
Met	12	0.29 (0.16, 0.41)	9	0.24 (0.12, 0.36)	14	0.2 (0.1, 0.3)
Orn	15	0.99 (0.49, 1.50)	15	0.73 (0.35, 1.1)	15	0.68 (0.4, 0.97)
P-SER	9	1.11 (0.05, 2.16)	12	0.49 (0.18, 0.81)	9	0.41 (0.19, 0.63)
PEA	13	0.53 (0.15, 0.91)	11	0.68 (0.27, 1.09)	13	0.43 (0.23, 0.64)
Phe	15	0.90 (0.60, 1.20)	14	0.67 (0.36, 0.99)	15	0.69 (0.46, 0.93)
Pro	**15**	**7.51 (5.82, 9.21)[a]**	**15**	**5.53 (4.2, 6.86)[b]**	**15**	**5.60 (4.28, 6.91)[b]**
Ser	**15**	**1.61 (1.11, 2.11)[a]**	**14**	**1.06 (0.66, 1.47)[b]**	**14**	**1.41 (1.01, 1.82)[ab]**
Tau	10	0.75 (0.29, 1.20)	9	0.73 (0.35, 1.11)	12	0.71 (0.43, 0.99)
Thr	15	1.27 (0.86, 1.67)	13	0.9 (0.51, 1.3)	15	1.1 (0.72, 1.48)
Trp	13	0.37 (0.23, 0.51)	14	0.33 (0.17, 0.49)	13	0.31 (0.18, 0.44)
Tyr	13	0.48 (0.35, 0.62)	9	0.48 (0.2, 0.77)	13	0.41 (0.25, 0.57)
Val	14	1.67 (0.76, 2.59)	12	1.34 (0.5, 2.18)	13	1.45 (0.92, 1.98)

C_{max}: Concentration at highest peak. P-EAA: (Plasma) essential amino acids; P-NEAA: (Plasma) non-essential amino acids; P-T21AA: (Plasma) total amino acids, all as defined in Section 2.6. Abbreviations of amino acids were used in the table. Data is expressed as mean (95% CI) in µmol·L^{-1}·g TAA^{-1}. Participants with missing peaks for individual amino acids were omitted. Pairwise comparisons between interventions were performed using linear mixed models with Bonferroni correction. Only amino acids with at least one significant pairwise difference between interventions were annotated and bolded, with values not sharing a common superscript letter differing significantly at $P \leq 0.05$. No difference remained significant after BH-FDR correction.

Supplementary Table S11: Table of AUC from curated Wood curves and imputation

Amino Acid	AUC (µmol·min ·L^{-1} ·g TAA^{-1})		
	**Control**	**ABSG**	**FBSG**
P-T21AA	241.56 (84.02, 399.11)	142.14 (63.13, 221.14)	499.03 (-203.49, 1201.55)
P-EAA	65.68 (10.37, 120.98)	42.28 (30.72, 53.85)	46.11 (20.11, 72.11)
P-NEAA	229.37 (118.97, 339.77)	122.41 (49.00, 195.83)	131.44 (60.87, 202.00)
α-ABA	1.97 (-0.10, 4.04)	6.90 (-4.10, 17.91)	1.30 (0.47, 2.12)
Ala	117.38 (63.01, 171.76)	83.84 (32.16, 135.52)	72.73 (48.80, 96.67)
Arg	8.59 (4.36, 12.82)	6.91 (0.66, 13.15)	7.19 (3.26, 11.12)
Asn	5.78 (2.14, 9.41)	3.38 (2.25, 4.50)	3.93 (1.41, 6.45)
Asp	0.26 (0.20, 0.31)	0.24 (0.21, 0.27)	0.21 (0.19, 0.24)
Cit	1.50 (0.96, 2.04)	1.23 (0.79, 1.67)	2.23 (1.11, 3.35)
Cys	2.33 (1.45, 3.21)	1.73 (1.51, 1.94)	2.03 (1.27, 2.79)
Gln	44.49 (27.16, 61.82)	56.99 (0.20, 113.77)	31.50 (20.64, 42.36)
Glu	6.64 (4.88, 8.39)	6.46 (3.30, 9.61)	5.80 (3.95, 7.64)
Gly	11.01 (4.29, 17.72)	14.33 (-0.07, 28.72)	11.54 (-0.23, 23.31)
His	5.19 (2.20, 8.17)	3.89 (1.35, 6.43)	3.26 (0.63, 5.88)
Ile	8.27 (-0.41, 16.95)	4.87 (2.34, 7.39)	4.58 (1.54, 7.61)
Leu	13.35 (0.05, 26.64)	7.89 (4.92, 10.87)	7.82 (3.08, 12.57)
Lys	5.41 (3.85, 6.97)	4.97 (3.78, 6.15)	5.36 (4.00, 6.72)
Met	2.55 (0.53, 4.57)	1.90 (0.57, 3.24)	1.36 (0.56, 2.16)
Orn	8.13 (1.55, 14.71)	5.01 (2.16, 7.86)	6.03 (2.18, 9.87)
P-SER	1.47 (0.90, 2.05)	1.18 (0.81, 1.55)	1.06 (0.87, 1.25)
PEA	1.67 (1.00, 2.35)	1.56 (1.03, 2.08)	1.18 (0.99, 1.37)
Phe	7.13 (3.18, 11.09)	5.34 (1.55, 9.14)	4.54 (2.40, 6.67)
Pro	85.99 (62.56, 109.42)	65.63 (36.58, 94.68)	56.43 (37.67, 75.18)
Ser	11.19 (3.40, 18.97)	7.60 (1.93, 13.27)	7.83 (1.47, 14.20)
Tau	2.49 (1.87, 3.12)	2.11 (1.25, 2.97)	1.83 (1.53, 2.14)
Thr	8.07 (3.79, 12.35)	7.33 (0.78, 13.88)	5.86 (2.27, 9.44)
Trp	1.61 (1.29, 1.92)	1.64 (1.07, 2.21)	1.48 (1.11, 1.84)
Tyr	2.99 (2.15, 3.83)	2.81 (2.08, 3.55)	2.58 (1.75, 3.40)
Val	6.94 (5.24, 8.64)	6.11 (4.25, 7.97)	5.69 (3.89, 7.49)

P-EAA: (Plasma) essential amino acids; P-NEAA: (Plasma) non-essential amino acids; P-T21AA: (Plasma) total amino acids, all as defined in Section 2.6. Abbreviations of amino acids were used in the table. AUC were calculated from accepted Wood curve fits, with excluded fits imputing values from raw data. Baseline of AUC was accounted for during fitting. Values are represented as mean (95% CI) in µmol·min·L^{-1}·g TAA^{-1}. Pairwise

comparisons between interventions were performed using linear mixed models with Bonferroni correction. No significant values were found.

Supplementary Table S12: Table of peak time (T_{max}) from curated Wood fits

Amino Acid	T_{max} (min) for Wood fits					
	Control		ABSG		FBSG	
	n	Average (95% CI)	n	Average (95% CI)	n	Average (95% CI)
P-T21AA	13	63.46 (47.07, 79.86)	11	46.67 (21.65, 71.69)	12	54.17 (39.20, 69.14)
P-EAA	10	52.99 (30.16, 75.82)	5	47.87 (33.36, 62.38)	10	50.32 (39.34, 61.30)
P-NEAA	13	84.43 (71.53, 97.35)	6	66.99 (32.59, 101.38)	11	64.06 (53.16, 74.96)
α-ABA	11	52.28 (32.68, 71.88)	13	66.39 (43.02, 89.76)	11	48.55 (32.04, 65.06)
Ala	14	90.92 (78.83, 103)	11	81.67 (56.26, 107.09)	13	77.32 (65.26, 89.37)
Arg	7	79.66 (48.29, 111.02)	7	84.58 (47.68, 121.48)	9	75.2 (48.93, 101.47)
Asn	**12**	**64.48 (45.56, 83.40)**[a]	**6**	**48.93 (28.08, 69.79)**[b]	**10**	**51.41 (29.72, 73.09)**[ab]
Cit	4	53.35 (-29.82, 136.52)	7	59.67 (28.82, 90.53)	8	103.52 (90.38, 116.67)
Cys	8	35.81 (20.79, 50.83)	5	27.95 (-6.62, 62.52)	7	34.8 (14.98, 54.61)
Gln	12	77.14 (59.02, 95.27)	6	81.48 (48.77, 114.19)	10	56.06 (37.13, 74.99)
Glu	**4**	**34.23 (-8.57, 77.03)**[a]	**8**	**48.62 (23.98, 73.25)**[b]	**4**	**38.60 (-2.06, 79.25)**[ab]
Gly	9	57.91 (28.56, 87.27)	6	82.76 (51.31, 114.2)	8	53.37 (24.26, 82.48)
His	12	64.83 (51.44, 78.22)	8	69.93 (45.04, 94.82)	12	52.57 (36.05, 69.09)
Ile	13	51.14 (35.26, 67.02)	10	54.72 (31.28, 78.17)	12	49.16 (38.64, 59.67)
Leu	12	49.42 (31.89, 66.95)	10	51.11 (29.96, 72.25)	12	44.64 (34.35, 54.94)
Lys	9	34.99 (18.23, 51.75)	4	33.3 (6.48, 60.12)	9	45.57 (29.07, 62.07)
Met	10	63.70 (40.78, 86.61)	7	57.67 (23.68, 91.65)	11	51.26 (37.13, 65.39)
Orn	14	58.73 (42.85, 74.61)	12	56.36 (39.74, 72.97)	9	58.27 (29.97, 86.56)
P-SER	3	10.54 (9.58, 11.50)	5	45.3 (24.12, 66.47)	4	19.95 (9.37, 30.54)
PEA	**9**	**61.18 (43.05, 79.32)**[a]	**5**	**33.65 (-11.34, 78.63)**[b]	**5**	**37.73 (20.53, 54.93)**[a]
Phe	13	64.86 (50.39, 79.33)	9	84.55 (62.58, 106.52)	14	59.4 (44.93, 73.88)
Pro	15	93.26 (82.68, 103.84)	15	92.84 (79.75, 105.93)	12	89.57 (75.26, 103.89)
Ser	14	52.18 (37.16, 67.2)	14	59.19 (37.78, 80.6)	12	49.79 (31.94, 67.64)
Tau	3	34.28 (-42.48, 111.04)	4	14.59 (4.07, 25.11)	7	32.36 (22.14, 42.58)
Thr	**12**	**58.15 (37.22, 79.08)**[a]	**10**	**48.42 (27.01, 69.83)**[ab]	**14**	**45.39 (34.23, 56.55)**[b]
Trp	11	45.81 (29.73, 61.89)	10	49.47 (33.79, 65.15)	11	36.83 (26.31, 47.34)
Tyr	10	46.71 (33.74, 59.68)	6	41.35 (24.81, 57.89)	12	49.06 (31.74, 66.37)
Val	9	42.76 (26.16, 59.36)	7	50.36 (20.68, 80.04)	10	46.82 (33.83, 59.82)

T_{max}: Time to peak concentration. P-EAA: (Plasma) essential amino acids; P-NEAA: (Plasma) non-essential amino acids; P-T21AA: (Plasma) total amino acids, all as defined in Section 2.6. Abbreviations of amino acids were used in the table. Data is expressed as mean (95% CI) in minutes. Participants with missing peaks after rejecting Wood fits for individual amino acids were omitted. Asp was not included in the table as curve was only successfully fitted in 7/45 records. Pairwise comparisons between interventions were performed using linear mixed models with Bonferroni correction. Only amino acids with at least one significant pairwise difference between interventions were annotated and bolded, with values not sharing a common superscript letter differing significantly at $P \leq 0.05$.

.

Supplementary Table S13: Table of highest peak concentration (C_{max}) from curated Wood curves and imputation

Amino Acid	C_{max} (µmol·L⁻¹·g TAA⁻¹)		
	Control	ABSG	FBSG
P-T21AA	118.10 (32.95, 203.25)	61.29 (6.60, 115.97)	62.98 (12.81, 113.14)
P-EAA	24.86 (3.03, 46.70)	15.25 (2.44, 28.06)	15.00 (1.85, 28.16)
P-NEAA	**155.50 (95.83, 215.17)**[a]	**89.49 (55.44, 123.55)**[ab]	**76.09 (33.58, 118.58)**[b]
α-ABA	0.65 (0.11, 1.19)	0.80 (0.27, 1.34)	0.62 (0.12, 1.13)
Ala	**42.43 (27.40, 57.45)**[a]	**27.86 (15.72, 40.00)**[ab]	**24.73 (15.36, 34.10)**[b]
Arg	4.20 (2.58, 5.83)	3.59 (1.99, 5.18)	3.56 (1.90, 5.22)
Asn	2.19 (0.88, 3.50)	2.18 (1.28, 3.08)	1.85 (0.92, 2.79)
Asp	0.10 (0.06, 0.13)	0.11 (0.08, 0.13)	0.08 (0.06, 0.10)
Cit	0.49 (0.35, 0.63)	0.43 (0.31, 0.55)	0.97 (0.27, 1.68)
Cys	0.70 (0.44, 0.97)	0.75 (0.24, 1.25)	1.34 (0.21, 2.47)
Gln	34.80 (12.91, 56.69)	20.47 (7.92, 33.01)	14.80 (2.59, 27.01)
Glu	2.35 (1.66, 3.03)	2.17 (1.48, 2.86)	2.18 (1.46, 2.90)
Gly	3.37 (0.06, 6.69)	4.23 (0.17, 8.29)	3.93 (0.09, 7.76)
His	2.85 (0.90, 4.80)	1.83 (0.36, 3.29)	1.04 (0.09, 1.99)
Ile	1.90 (0.26, 3.55)	1.89 (0.62, 3.15)	1.50 (0.45, 2.55)
Leu	3.61 (0.56, 6.66)	3.52 (1.15, 5.90)	2.75 (0.75, 4.76)
Lys	1.78 (1.42, 2.15)	1.52 (1.16, 1.88)	1.57 (1.27, 1.86)
Met	0.69 (0.19, 1.18)	0.72 (0.22, 1.22)	0.42 (0.13, 0.70)
Orn	2.99 (0.90, 5.09)	2.05 (0.66, 3.44)	2.23 (0.75, 3.71)
P-SER	0.81 (0.52, 1.11)	0.62 (0.43, 0.82)	0.58 (0.46, 0.71)
PEA	**0.88 (0.58, 1.18)**[a]	**0.79 (0.52, 1.05)**[ab]	**0.56 (0.43, 0.70)**[b]
Phe	3.11 (1.44, 4.78)	2.49 (1.02, 3.96)	2.10 (0.80, 3.40)
Pro	**24.93 (19.64, 30.21)**[a]	**15.87 (10.53, 21.21)**[b]	**17.16 (12.92, 21.40)**[b]
Ser	4.22 (0.99, 7.45)	2.57 (0.33, 4.81)	2.84 (0.39, 5.29)
Tau	1.36 (1.00, 1.72)	1.11 (0.65, 1.58)	1.01 (0.79, 1.24)
Thr	2.99 (0.66, 5.31)	2.49 (0.17, 4.81)	1.94 (0.40, 3.47)
Trp	0.64 (0.15, 1.13)	0.60 (0.16, 1.04)	0.55 (0.10, 1.00)
Tyr	1.00 (0.39, 1.61)	0.64 (0.54, 0.74)	0.65 (0.54, 0.75)
Val	2.21 (1.84, 2.58)	3.45 (0.10, 6.81)	1.83 (1.47, 2.19)

C_{max}: Concentration at highest peak. P-EAA: (Plasma) essential amino acids; P-NEAA: (Plasma) non-essential amino acids; P-T21AA: (Plasma) total amino acids, all as defined in Section 2.6. Abbreviations of amino acids were used in the table. Data is expressed as mean (95% CI) in (µmol·L⁻¹·g TAA⁻¹). Peak concentrations were

calculated from accepted Wood curve fits, with excluded fits imputing values from raw data. Only amino acids with at least one significant pairwise difference between interventions were annotated and bolded, with values not sharing a common superscript letter differing significantly at $P \leq 0.05$.

Supplementary Table S14: Summary of curated Wood curves accepted rates

Amino Acid or Grouped Totals	Rejected (n)	Accepted (n)	Rejected (%)	Accepted (%)
**Individual Amino Acids**				
Threonine	9	36	20.0	80.0
Valine	19	26	42.2	57.8
Methionine	17	28	37.8	62.2
Isoleucine	10	35	22.2	77.8
Leucine	11	34	24.4	75.6
Phenylalanine	9	36	20.0	80.0
Tryptophan	13	32	28.9	71.1
Lysine	23	22	51.1	48.9
Histidine	13	32	28.9	71.1
Serine	5	40	11.1	88.9
Asparagine	17	28	37.8	62.2
Glutamic acid	29	16	64.4	35.6
Glutamine	17	28	37.8	62.2
Glycine	22	23	48.9	51.1
Alanine	7	38	15.6	84.4
Cysteine	25	20	55.6	44.4
Tyrosine	17	28	37.8	62.2
Arginine	22	23	48.9	51.1
Proline	6	39	13.3	86.7
Aspartic acid	38	7	84.4	15.6
O-phospho-L-Serine	33	12	73.3	26.7
Taurine	31	14	68.9	31.1
O-phospho-ethanolamine	26	19	57.8	42.2
Citrulline	26	19	57.8	42.2
α-amino-n-butyric	10	35	22.2	77.8
Ornithine	10	35	22.2	77.8
**Grouped Totals**				
P-T21AA	9	36	20.0	80.0
P-EAA	20	25	44.4	55.6
P-NEAA	15	30	33.3	66.7
**Total Curation:**	**509**	**796**	**39.0**	**61.0**

P-EAA: (Plasma) essential amino acids; P-NEAA: (Plasma) non-essential amino acids; P-T21AA: (Plasma) total amino acids, all as defined in Section 2.6.

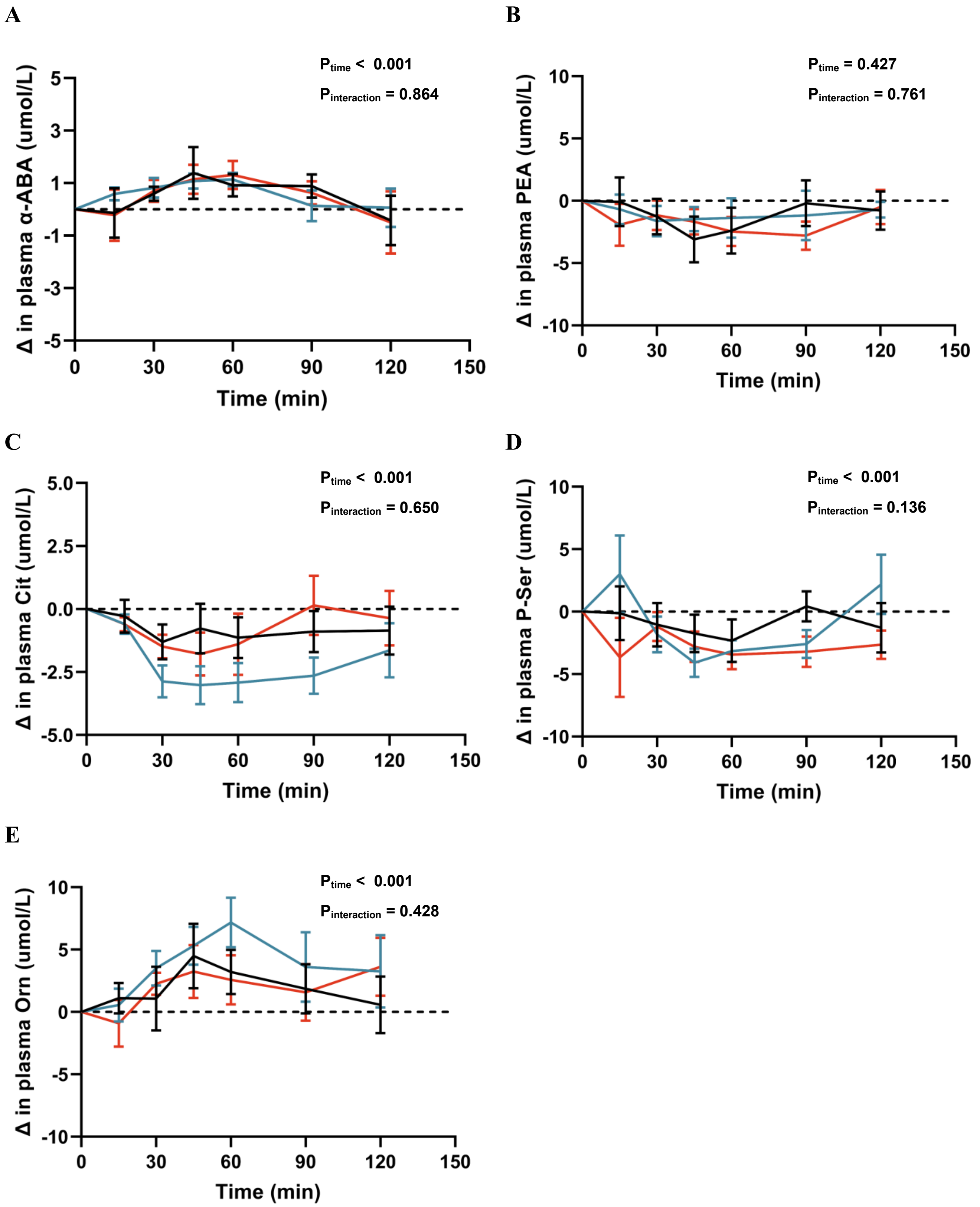

Supplementary Figure S1: Change in non-proteinogenic AA in blood plasma.
Blue indicates Control, **Black** indicates ABSG and **Red** indicates FBSG. Changes from baseline of (A)) α-amino-n-butyric. (B) O-phospho-ethanolamine. (C) Citrulline. (D) O-phospho-L-Serine. (E) Ornithine. P-values

determined with linear mixed model with Bonferroni correction showed no significant interaction effect and intervention effect, but significant time effect ($P \leq 0.05$) for all but (B).

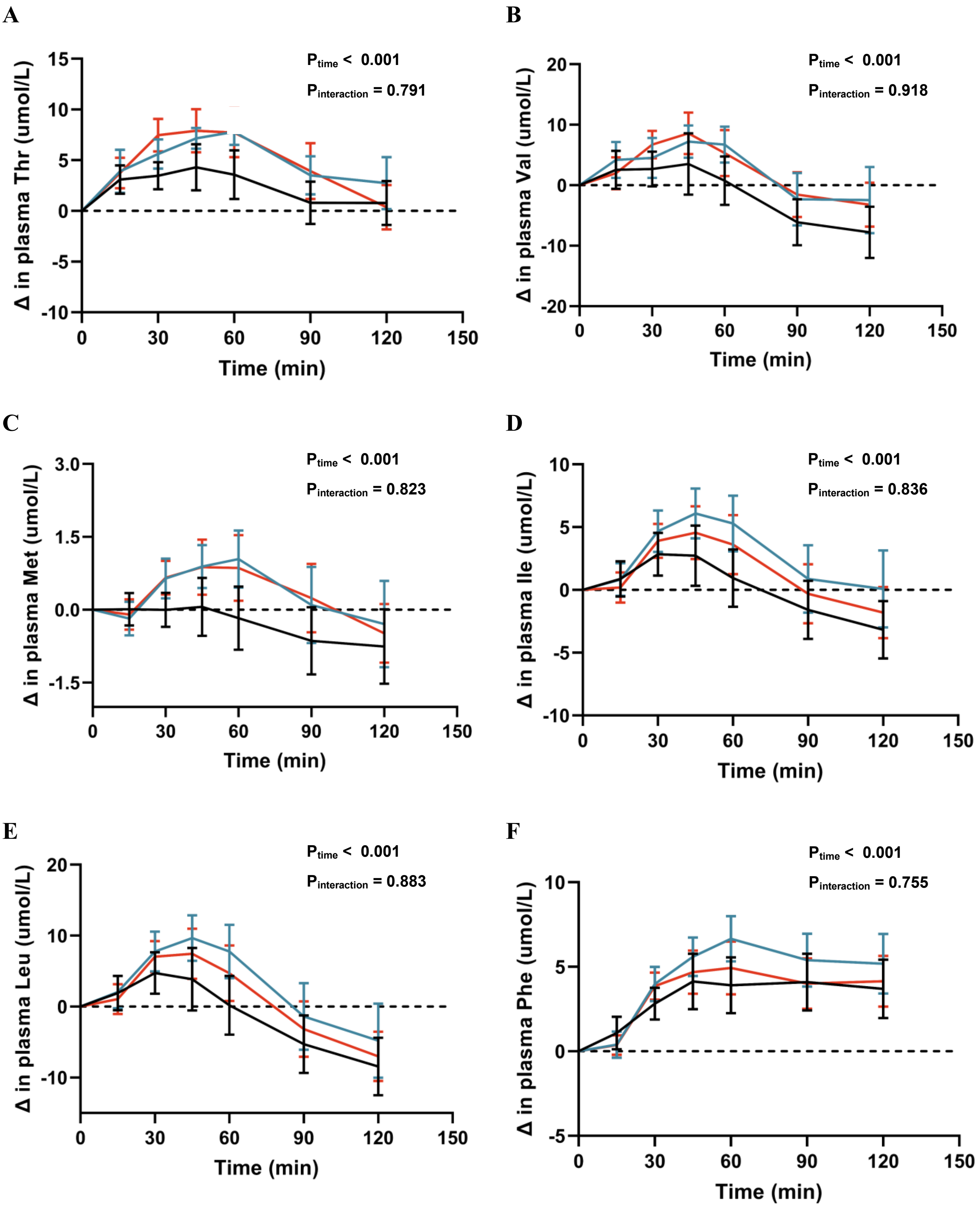
A
P_{time} < 0.001
$P_{interaction}$ = 0.791
Δ in plasma Thr (umol/L)
Time (min)
B
P_{time} < 0.001
$P_{interaction}$ = 0.918
Δ in plasma Val (umol/L)
Time (min)
C
P_{time} < 0.001
$P_{interaction}$ = 0.823
Δ in plasma Met (umol/L)
Time (min)
D
P_{time} < 0.001
$P_{interaction}$ = 0.836
Δ in plasma Ile (umol/L)
Time (min)
E
P_{time} < 0.001
$P_{interaction}$ = 0.883
Δ in plasma Leu (umol/L)
Time (min)
F
P_{time} < 0.001
$P_{interaction}$ = 0.755
Δ in plasma Phe (umol/L)
Time (min)

G

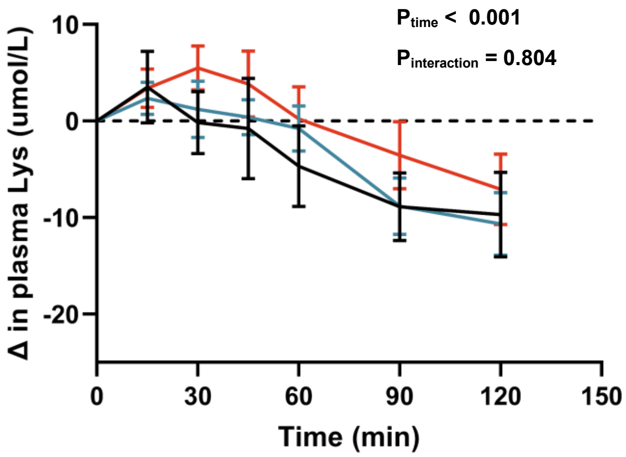

H

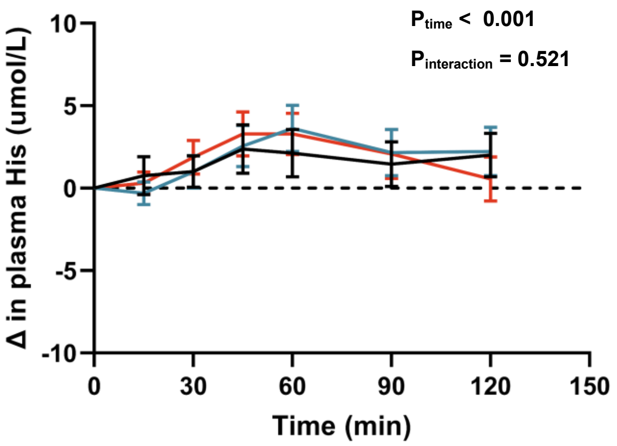

I

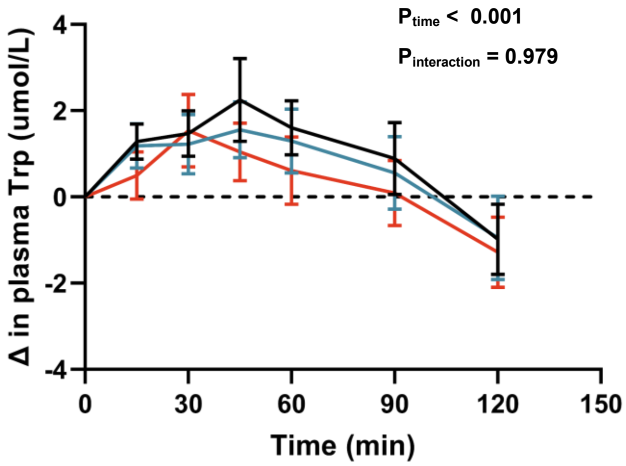

Supplementary Figure S2: Change in EAA in blood plasma .
Blue indicates Control, **Black** indicates ABSG and **Red** indicates FBSG. Changes from baseline of (A) Threonine. (B) Valine. (C) Methionine. (D) Isoleucine. (E) Leucine. (F) Phenylalanine. (G) Lysine. (H)

Histidine. (I) Tryptophan. P-values determined with linear mixed model with Bonferroni correction showed no significant interaction effect and intervention effect, but significant time effect ($P \leq 0.05$).

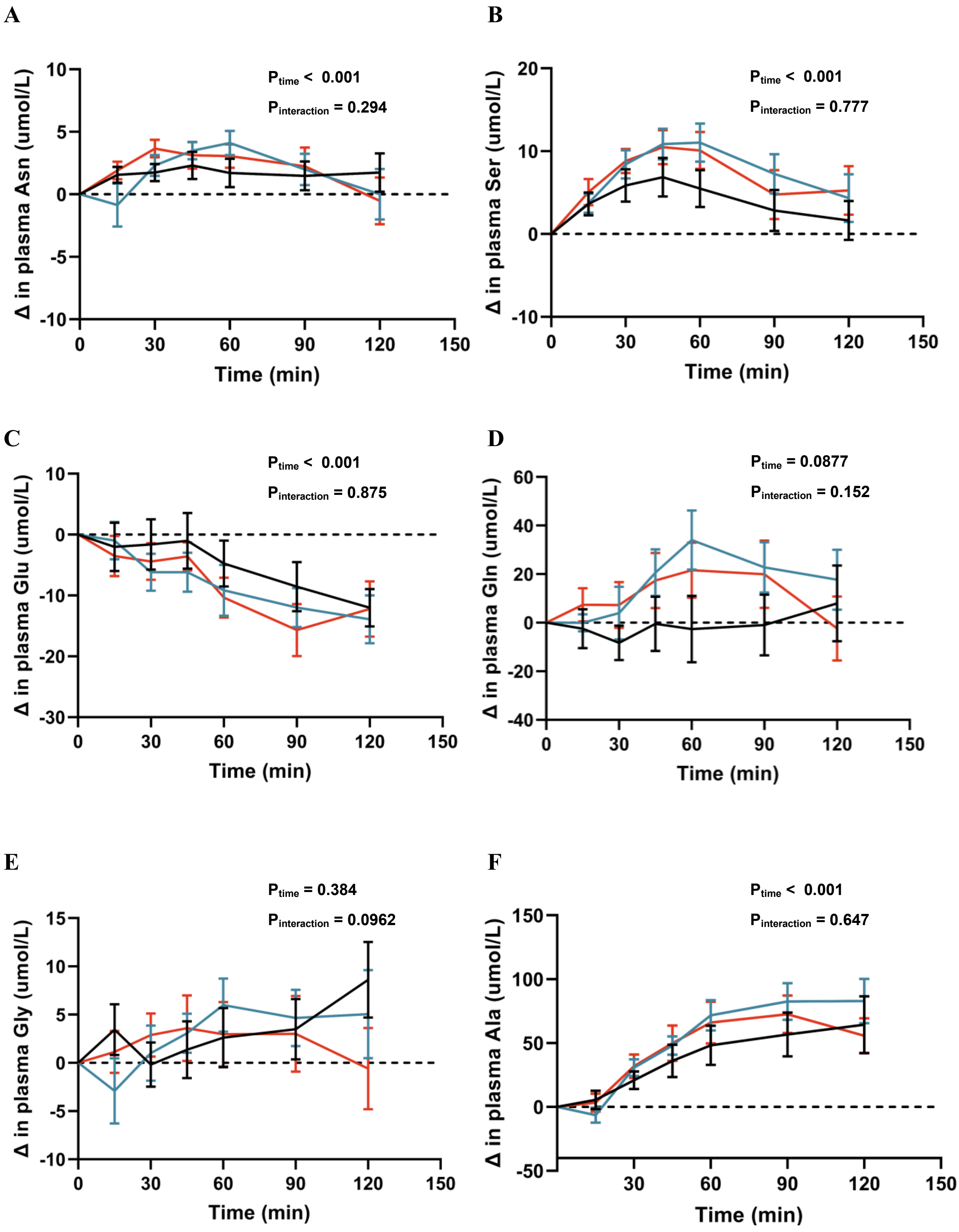
A
P_time < 0.001
P_interaction = 0.294
Δ in plasma Asn (umol/L)
Time (min)
B
P_time < 0.001
P_interaction = 0.777
Δ in plasma Ser (umol/L)
Time (min)
C
P_time < 0.001
P_interaction = 0.875
Δ in plasma Glu (umol/L)
Time (min)
D
P_time = 0.0877
P_interaction = 0.152
Δ in plasma Gln (umol/L)
Time (min)
E
P_time = 0.384
P_interaction = 0.0962
Δ in plasma Gly (umol/L)
Time (min)
F
P_time < 0.001
P_interaction = 0.647
Δ in plasma Ala (umol/L)
Time (min)

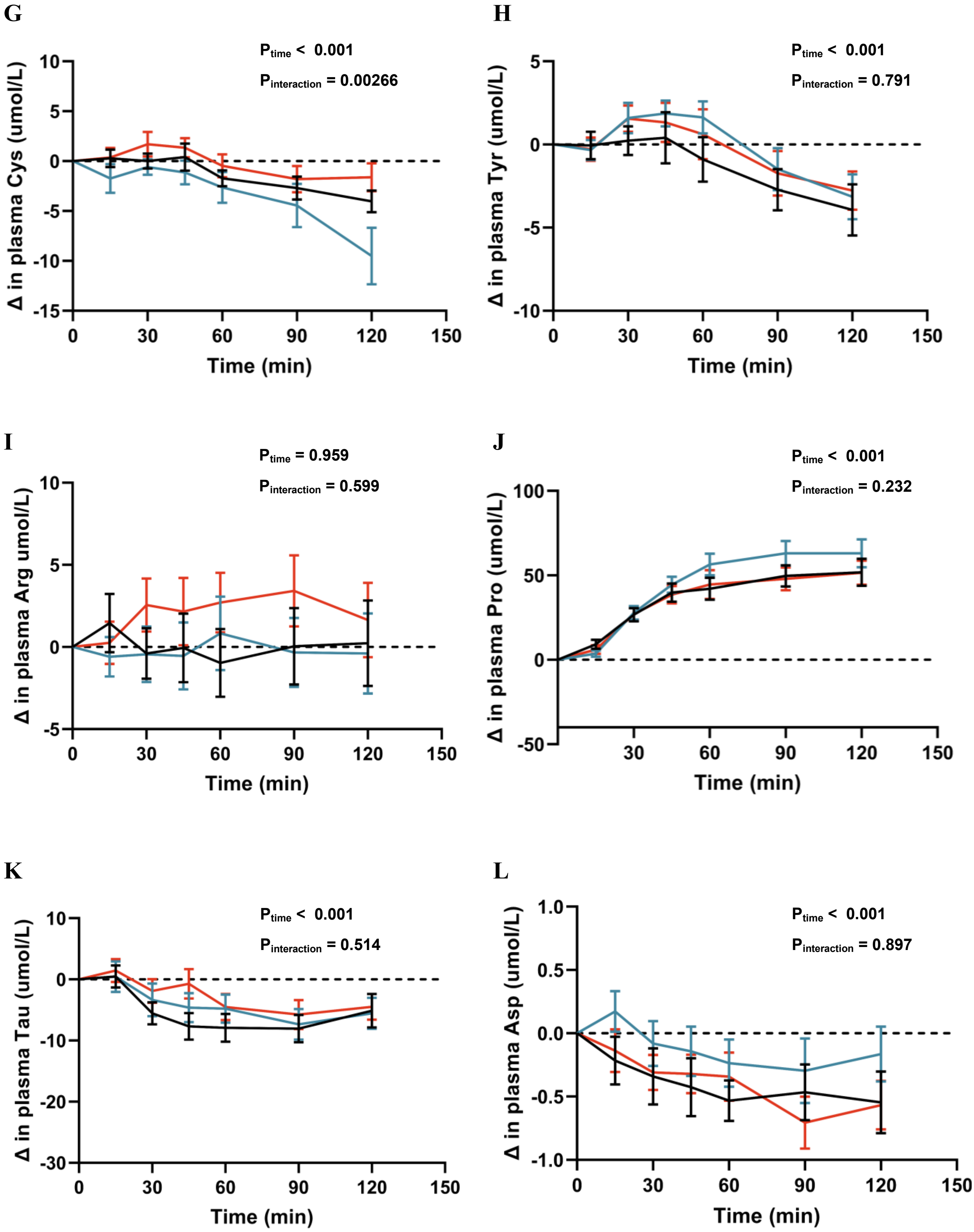
G
P time < 0.001
P interaction = 0.00266
Δ in plasma Cys (umol/L)
Time (min)
H
P time < 0.001
P interaction = 0.791
Δ in plasma Tyr (umol/L)
Time (min)
I
P time = 0.959
P interaction = 0.599
Δ in plasma Arg umol/L)
Time (min)
J
P time < 0.001
P interaction = 0.232
Δ in plasma Pro (umol/L)
Time (min)
K
P time < 0.001
P interaction = 0.514
Δ in plasma Tau (umol/L)
Time (min)
L
P time < 0.001
P interaction = 0.897
Δ in plasma Asp (umol/L)
Time (min)

M

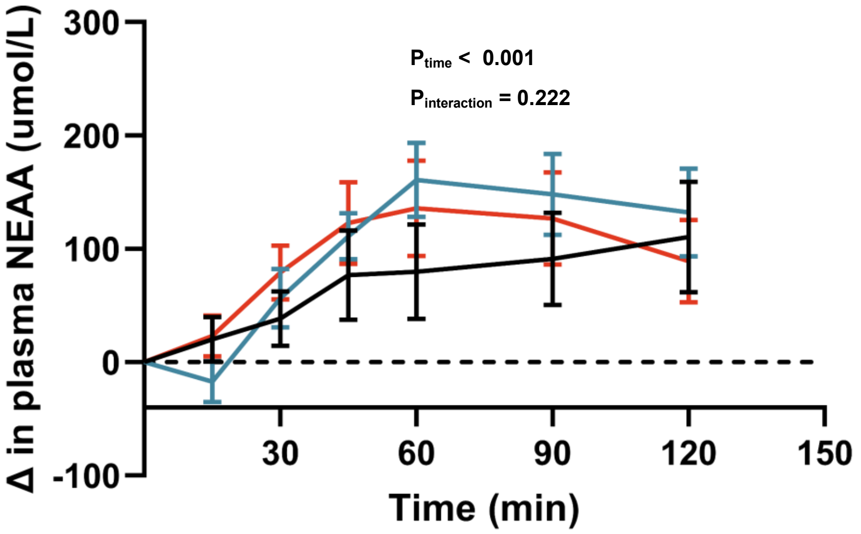

Supplementary Figure S3: Change in NEAA in blood plasma.

Blue indicates Control, **Black** indicates ABSG and **Red** indicates FBSG. Changes from baseline of (A) Asparagine. (B) Serine. (C) Glutamic Acid. (D) Glutamine. (E) Glycine. (F) Alanine. (G) Cysteine. (H) Tyrosine. (I) Arginine. (J) Proline. (K) Taurine. (L) Aspartic Acid. (M) P-NEAA. P-values determined with linear mixed model with Bonferroni correction showed significant interaction effect only on (G).
